# Fouling Control of Ion-Selective Electrodes (ISEs) in Aquatic and Aquacultural Environments: A Comprehensive Review

**DOI:** 10.3390/s25247515

**Published:** 2025-12-10

**Authors:** Patrick Rinn, Fabian Boruta, Peter Czermak, Mehrdad Ebrahimi

**Affiliations:** 1 Institute of Bioprocess Engineering and Pharmaceutical Technology (IBPT), University of Applied Sciences Mittelhessen (THM), Wiesenstraße 14, 35390 Giessen, Germany; patrick.rinn@lse.thm.de (P.R.); peter.czermak@lse.thm.de (P.C.); 2 Straubtec GmbH & Co. KG, 63877 Sailauf, Germany; fabian.boruta@straubtec.de

**Keywords:** ion-selective electrode (ISE), aquaculture, fouling mechanisms, fouling detection, fouling control, fouling prevention, antifouling strategies

## Abstract

**Highlights:**

**What are the main findings?**

- Ion-selective electrodes (ISEs) are susceptible to fouling, which affects their sensitivity, accuracy, and operational lifetime.
- The review consolidates fouling mechanisms with available detection, cleaning, and antifouling strategies, providing a unified overview of their effectiveness and limitations.

**What are the implications of the main findings?**

- Antifouling strategies are essential to ensure the accurate and long-term operation of ISEs.
- Combining physical, chemical, and material-based approaches is crucial to effectively combat fouling phenomena.

**Abstract:**

Real-time monitoring is essential for maintaining water quality and optimizing aquaculture productivity. Ion-selective electrodes (ISEs) are widely used to measure key parameters such as pH, nitrate, and dissolved oxygen in aquatic environments. However, these sensors are prone to fouling, the non-specific adsorption of organic, inorganic, and biological matter, which leads to potential drift (e.g., 1–10 mV/h), loss of sensitivity (e.g., ~40% in 20 days), and reduced lifespan (e.g., 3 months), depending on membrane formulation and environmental conditions. This review summarizes current research from mostly the last two decades with around 150 scientific studies on fouling phenomena affecting ISEs, as well as recent advances in fouling detection, cleaning, and antifouling strategies. Detection methods range from electrochemical approaches such as potentiometry and impedance spectroscopy to biochemical, chemical, and spectroscopic techniques. Regeneration and antifouling strategies combine mechanical, chemical, and material-based approaches to mitigate fouling and extend sensor longevity. Special emphasis is placed on environmentally safe antifouling coatings and material innovations applicable to long-term monitoring in aquaculture systems. The combination of complementary antifouling measures is key to achieving accurate, stable, and sustainable ISE performance in complex water matrices.

## 1. Introduction

### 1.1. Literature Regarding the Topic

Verma et al. (2022) and Lindholm-Lehto (2023) provide a comprehensive overview on water quality parameters used for aquaculture, including temperature, dissolved oxygen, salinity, and turbidity [1,2]. Additional information regarding the real-time monitoring of these parameters is given by Lindholm-Lehto (2023) [2]. A general overview of antifoulants used in aquatic environments is given by Parisi et al. (2022) [3], and information on antifoulants used specifically for ion-selective electrodes (ISEs) can be found given by Qi et al. (2022) [4] and Liu et al. (2025) [5]. However, these studies treat fouling mechanisms, detection techniques, cleaning strategies, and antifouling approaches largely separately. To our knowledge, none of the existing reviews combine these aspects into a comprehensive, application-focused approach for long-term deployment of ISEs in aquatic or aquaculture systems. Publications from 2000 to 2025 with emphasis on the last two years were chosen from Google Scholar, with selected keywords regarding ion-selective electrodes, fouling, drift, cleaning, antifouling, and aquaculture. Studies were included when they addressed explicit keywords and excluded when lacking the methodological relevance or explicit discussion of fouling effects. The following section outlines the concept of water quality to understand challenges regarding fouling on ISEs.

### 1.2. Water Quality

Water quality serves as a key indicator of environmental integrity and ecosystem stability. It reflects the combined effects of natural processes and anthropogenic influences, including industrialization, agricultural runoff, and urbanization [6,7,8]. Understanding and controlling the defining parameters of water quality are therefore fundamental prerequisites for effective monitoring, pollution prevention, and sustainable resource management. Monitoring programmes based on standardized water quality parameters enable the early detection of pollution events and the assessment of remediation strategies. These parameters act as diagnostic indicators that capture both the current state and temporal trends of aquatic environments, thereby supporting water management decisions and ensuring compliance with environmental regulations [6].

#### Global Context and Importance of Water Quality

The availability or scarcity of freshwater is a major global challenge that increasingly threatens human existence [6,7]. Approximately one third of the world’s drinking water is obtained from surface waters such as rivers, lakes (including dam reservoirs), and canals. These also serve as sinks for the discharge of domestic and industrial wastewater. The disposal of untreated or partially treated wastewater is a worldwide issue and represents a major source of water pollution affecting freshwater bodies [8]. To develop effective strategies for reducing and eliminating pollution, specific water quality parameters must be monitored and maintained within defined limits. These parameters and their continuous monitoring have a significant impact on (i) human well-being (e.g., health, property, access to drinking water) [9], (ii) the environment (e.g., eutrophication, suppression of photosynthetic systems, dam functionality, aquatic ecosystem health) [10,11], and (iii) industrial applications (e.g., dairy, textile, and other wastewater processes) [12,13,14]. In aquaculture, water quality is critical. It directly determines animal health and production efficiency.

### 1.3. Relevance of Water Quality for Aquaculture

Maintaining good water quality is essential for aquaculture production, as aquatic organisms are directly dependent on their surrounding water for respiration, nutrition, and growth. The industrial cultivation of fish, shellfish, aquatic plants, and other marine organisms such as shrimp in freshwater, saltwater, or brackish environments for human consumption is defined as aquaculture [15]. Reducing inputs (e.g., feed), optimizing outputs, and minimizing pollution make aquaculture an attractive and sustainable alternative to conventional fishing [16]. Aquaculture is one of the fastest-growing food-producing sectors worldwide, now surpassing capture fisheries and accounting for approximately 53% of total production for human consumption [17]. Similarly to agriculture, aquaculture has undergone significant technological transformation in recent decades to meet growing global demands [17]. Ensuring and controlling water quality is crucial for achieving high productivity and maintaining healthy stock. Elevated mortality rates are often linked to poor water quality [18]. Continuous monitoring of physicochemical parameters can reduce production losses by 20–40% [17]. The strong correlation between water quality and the physiological health of cultured organisms means that deviations from optimal conditions can lead to stress, disease, or death [19]. Consequently, precise control of key parameters is vital to sustain both productivity and food quality in aquaculture systems. These critical parameters and their acceptable limits vary widely between species [17] and are particularly complex in multi-species systems [20]. Therefore, real-time monitoring is required to support informed decision-making by operators and prevent water quality deterioration before it impacts animal welfare.

Additionally, water quality can differ significantly between surface water and groundwater. Even local variations in tap or well water (depending on site location [18]) may require pretreatment to achieve optimal cultivation conditions [21]. Such variability underscores the importance of deploying robust, real-time monitoring systems, such as ion-selective electrodes, that can continuously track essential parameters like pH, salinity, or dissolved oxygen in aquaculture environments. Integrating these sensors into recirculating aquaculture systems (RAS) or other controlled setups ensures consistent environmental stability, minimizes fouling-related signal drift, and enhances automation for sustainable production. The key parameters of water quality control in aquaculture are listed in the following section.

### 1.4. Key Water Quality Parameters for Aquaculture

Key water quality parameters for aquaculture typically include the following:

- Temperature,
- Dissolved oxygen (DO),
- Colour/turbidity,
- Electrical conductivity (EC),
- Ozone or Oxidation–reduction potential (ORP),
- pH (pondus hydrogenii), and
- Specific ions or trace elements such as nitrate, sodium, and chloride [1,2].

**Figure 1:**
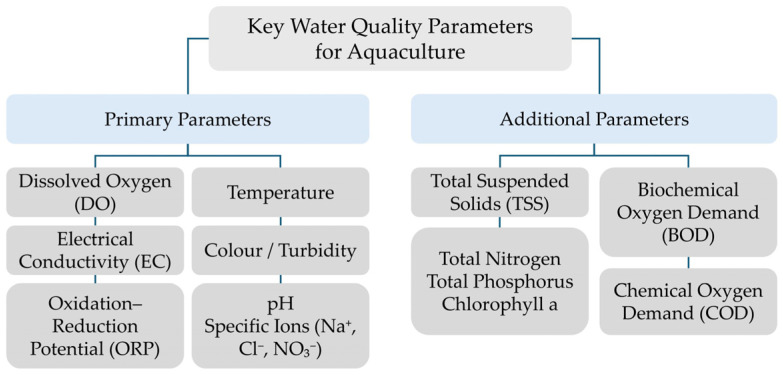
Overview of key water quality parameters for aquaculture.

These parameters jointly determine the chemical and biological balance of aquatic environments and directly influence the metabolism, growth, and reproduction of cultured species. Temperature and DO are key regulators of respiration and enzymatic activity, while pH and ORP define the redox conditions that control nutrient availability and ammonia toxicity [22]. Electrical conductivity, salinity, and the presence of dissolved ions (e.g., Na^+^, Cl^−^, NO_3_^−^) serve as indicators of the overall ionic composition, which affects osmoregulation and stress tolerance in aquatic organisms [22]. Additional relevant parameters include total suspended solids (TSS), biochemical oxygen demand (BOD), chemical oxygen demand (COD), total nitrogen, total phosphorus, and chlorophyll a (Figure 1) [23].

These indicators provide essential insights into organic load, eutrophication potential, and biological productivity in aquaculture systems. Elevated TSS and BOD values may reduce light penetration and oxygen availability, while increased COD reflects the accumulation of oxidizable organic matter. The concentrations of total nitrogen and phosphorus are critical for evaluating nutrient cycling and for preventing excessive algal growth [23]. Continuous monitoring of these parameters in aquaculture is primarily achieved through electrochemical and optical sensor systems, including thermistors, ion-selective electrodes (ISEs), Clark-type oxygen sensors, and nephelometric or conductivity probes. Integration of these sensors in RAS enables real-time control of water quality and early detection of fouling or chemical imbalance, ensuring stable operational conditions for aquatic organisms [22,23].

## 2. Real-Time Monitoring of Water Quality Parameters: Concepts and Applications

Real-time monitoring refers to the continuous observation and acquisition of data for specific parameters while a process is running [24]. It has been implemented in various industrial and environmental applications, such as health monitoring (e.g., heart disease, blood pressure, diabetes) [25], nutrient monitoring [26], bioreactors [27], wastewater treatment [28], and aquaculture [29]. Today, a wide variety of techniques are available for water quality monitoring, including sensors, analytical technologies (chromatography, mass spectrometry), biochemical and molecular methods (immunoassays, PCR assays, culture-based techniques), and biophysical technologies (biosensors and nano sensors) for detecting environmental contaminants [15]. A small comparison regarding their performance advantages and disadvantages is shown in Table 1.

## 3. Sensor Technologies for Water Quality Monitoring and Process Control

Sensor technologies play a fundamental role in advancing scientific understanding, process automation, and operational control. They enable timely intervention and corrective action in critical situations. For instance, precision aquaculture, based on sensor networks, can help minimize or prevent the negative environmental impacts of aquaculture activities [30]. Water quality sensors are used to evaluate process efficiency, optimize the performance of treatment and purification systems, and assess the ecological health of rivers, lakes, and wastewater streams. Despite their advantages, sensors are highly susceptible to fouling and biofouling in aqueous environments, which deteriorates sensor performance, increases maintenance requirements, degrades data quality, and consequently raises operational costs [31].

While sensors are widely used in terrestrial applications, their deployment in aquatic environments remains limited due to challenges such as high salinity (corrosion), fouling and biofouling, and the loss of waterproof integrity [16,30].

Kruse (2018) classifies chemical sensors into four main categories [32]:

1. Mechanical transduction sensors detect physical deformations, pressure, or flow variations and convert them into measurable signals, often applied in flow rate or turbidity monitoring.
2. Optical transduction sensors utilize light absorption, fluorescence, or scattering to determine parameters such as turbidity, colour, or dissolved organic matter.
3. Electrochemical transduction sensors convert chemical or ionic activities into electrical signals and include potentiometric, amperometric, and conductometric systems. Among these, ISEs are widely used for monitoring pH, ionic strength, and nutrient concentrations in aquatic environments.
4. Electrical transduction sensors measure variations in impedance, capacitance, or conductivity caused by chemical or biological interactions, offering high sensitivity in complex matrices.

A comparison of the sensor categories is presented in Table 2.

Among these, electrochemical sensors, redox-related parameters (e.g., DO, ORP), and particularly ISEs (pH and other ions) are the most widely used [32].

## 4. Ion-Selective Electrodes: Structure, Function, and Operational Principles

To achieve accurate, real-time monitoring of critical water quality parameters such as pH, nitrate, and other ions, specialized electrochemical sensors known as ISEs are employed. ISEs are designed to selectively detect target ions in aqueous solutions [33] and are widely used in various online monitoring applications worldwide to measure ions such as nitrate [34], titanium [35], lead [36], ammonium, lithium, sodium, potassium, caesium, silver, calcium, copper, cadmium, iron, cerium, chloride, sulphate, and phosphate [37]. They are increasingly integrated into automated systems, including titration setups and nutrient management platforms [38,39]. The selectivity is enabled by semi-permeable membranes, which allow the passage of only specific ions of interest [40].

### 4.1. General Structure and Working Principle

ISEs typically consist of two half-cells:

- a measuring half-cell, equipped with an ion-selective membrane that determines ion permeability, and
- a reference half-cell, usually containing an Ag/AgCl electrode.

**Figure 2:**
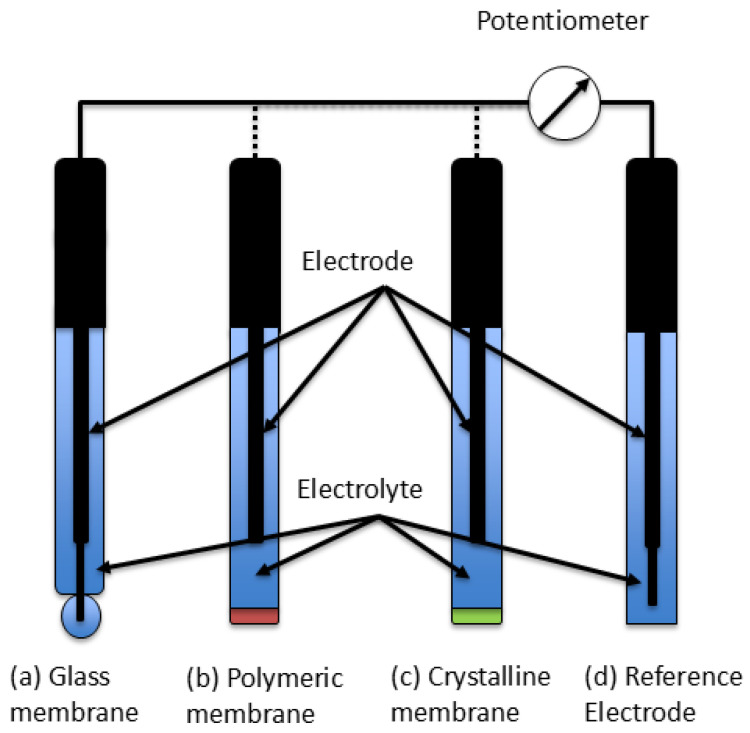
Working principle of a potentiometric ion-selective electrode, with a (**a**) glass membrane, (**b**) polymeric membrane, (**c**) crystalline/solid-contact membrane, and (**d**) reference electrode.

Each half-cell contains an electrode immersed in an electrolyte solution (e.g., KCl) [41]. The potential difference between the two electrodes follows the Nernst equation and is thus proportional to the ion concentration or activity [42]. A schematic representation of a typical ISE setup is shown in Figure 2.

Common ISEs integrate both half-cells into a single probe body (e.g., made of PEEK) with a sensing tip in contact with the sample solution [43].

Three main types of ISEs can be distinguished:

1. ISEs with a glass membrane (e.g., multi-component chalcogenide for Pb^2+^ selectivity),
2. ISEs with a polymeric ion-selective membrane, and
3. ISEs with a crystalline or solid-state ion-selective membrane [44].

### 4.2. Glass Membrane Ion-Selective Electrodes

A standard pH electrode (glass electrode) detects protons (H^+^) or hydronium ions (H_3_O^+^) using an ion-selective glass membrane [45]. The sensor measures a pH-dependent potential difference across the membrane when immersed in a solution. This potential difference between the measuring and reference electrodes is proportional to the pH value according to the Nernst equation [46]. The response time of an ISE is defined as the time between immersion of the sensing element in the sample and the moment when the potential reaches a steady state within ±1 mV (or 90% of its final value), and can be so short that the associated electronics become the limiting factor.

### 4.3. Polymeric Ion-Selective Membranes

Polymeric ion-selective membranes typically consist of the following components [47,48]:

1. Polymer matrix—provides mechanical stability and forms the base structure for other components (commonly poly(vinyl chloride), PVC);
2. Plasticizer—softens the polymer matrix and enhances ion mobility and flexibility (e.g., phthalates such as dioctyl phthalate);
3. Ionophore—an embedded ion carrier that binds specific ions, ensuring selectivity (e.g., quinazoline derivatives such as dibutyl(8-hydroxyquinolin-2-yl)methyl phosphonate); and
4. Ion exchanger/lipophilic additive—facilitates ion exchange, maintains electroneutrality, and improves membrane conductivity and selectivity (e.g., potassium tetrakis(p-chlorophenyl)borate).

### 4.4. Solid-State and All-Solid-Contact Ion-Selective Electrodes

Solid-state ISEs (e.g., based on supercapacitor materials such as nickel cobalt sulphide) do not contain a liquid electrolyte [49]. Consequently, they are easier to miniaturize, are not prone to evaporation, and exhibit reduced potential drift compared to conventional ISEs, since there is no volume change in the electrolyte. However, they can be sensitive to pressure and temperature variations [50]. In these sensors, ion-to-electron transduction occurs via solid contact between the ion-selective membrane and the electronically conductive substrate [50].

### 4.5. Construction and Sensor Body Materials

Beyond serving as a protective barrier against contamination (e.g., of the internal electrolyte) and shielding the half-cells, the sensor body material itself significantly influences measurement stability. Certain components (e.g., from a PVC body) may leach into the ion-selective membrane, reducing ion selectivity, and vice versa [51]. A trade-off must therefore be made: materials such as PTFE and PEEK offer excellent inertness and chemical resistance but poor membrane adhesion, whereas PVC provides superior adhesion but lower chemical resistance [51].

### 4.6. Laboratory vs. Field

A comparison of ISE-type sensors with colorimetric methods such as the phenate method and the salicylate method in aquacultures to determine the total ammonia nitrogen in RAS showed a less accurate correlation (R^2^ ranging from 0.919 to 0.996) for ISEs in comparison to the phenate and salicylate methods (R^2^ = 0.996), whilst ISEs provided larger detection rates (0.05–14,000 ppm for NH_3_ ISE and 0.04–14,000 ppm for NH_4_^+^ ISE in comparison to 0.02–5 ppm for Nessler’s reaction) without the use of chemicals/dyes which alter the medium (e.g., rise in pH) [52]. Furthermore, concentrations above 5 ppm exceed the limit of detection of the salicylate method (1 ppm), the phenate method (2 ppm), and Nessler’s reaction (5 ppm) [52], which are offline and hence geographically and temporally divided from the process.

Whilst most analytical methods provide offline data, sensors excel through their implementation inside the process and thus field deployment, hence providing real-time inline data. Accurate prediction of water quality parameters for improved management is a current challenge in the aquaculture industry [53]. Real-time monitoring generates large volumes and diverse types of data [17], which enable the anticipation and prediction of changes in water quality parameters [17]. Moreover, warnings and automated alarms can be established based on prior monitoring experience [16]. Further advantages and limitations of ISEs are stated in the following section.

### 4.7. Advantages and Limitations of ISEs

Potentiometric ISEs typically exhibit rapid response times, as fast as 3 s, and long operational lifetimes, e.g., up to two months for Fe^2+^-ISEs [54]. In general, ion-selective electrodes offer significant advantages in terms of accuracy, measurement speed, cost, and sensitivity compared to analytical techniques such as UV/VIS spectroscopy for detecting specific ionic activities on-site [55]. Modern ISEs are also cost-competitive with UV/VIS and UV sensors in applications such as activated sludge processes, where they monitor nitrate and nitrite concentrations and thus aid in aeration control [56].

Beyond analytical precision, ISEs also offer several practical advantages in real-time process monitoring. In comparison with optical or amperometric sensors, ISEs operate without reagents or gas-phase calibration. They are not influenced by colour or turbidity, consume minimal power, and can be easily miniaturized and integrated into multiparameter sensor arrays [55,56,57]. Their compact, robust design enables long-term in situ measurements in harsh aquatic environments, where high salinity or turbidity may interfere with optical systems. These features make ISEs particularly suitable for continuous monitoring and automated control of key parameters such as pH, nitrate, and ammonium in aquaculture and wastewater treatment processes [56,58]. Furthermore, broad selectivity of ionophores leads to a broad range of parameters covered by ISEs.

However, their long-term stability remains limited by membrane fouling, drift, and temperature sensitivity, necessitating periodic cleaning and recalibration [58,59]. Further limitations regarding ISEs are as follows:

- Unlike methods such as chromatography, ISEs detect only free ions (e.g., F^−^), reflecting ionic activity, while bound or complexed species (e.g., fluorides) remain undetected in complex solutions [60]. To provide better correlations regarding the ionic strength of the solution, ionic strength adjuster can be used prior to calibration, thus imitating field conditions.
- At high concentrations, increased ionic strength reduces the activity of the target ion, leading to a nonlinear relationship between activity and concentration [57]. Usage of the ISE in the linear range is usually stated by the producer as its operating range, which may be insufficient in salt water or industrial waters.
- Interference effects occuring from ions with similar charge and size—for instance, NO_3_^−^-ISEs are affected by IO_3_^−^ and I^−^ [61], while NH_4_^+^-ISEs suffer from H^+^ interference [62]. This effect is crucial for multi-ion systems such as aquacultures, saltwater, and wastewater, where cross-ion interferences falsify measurements. To address the problem, a cross-ion compensation via modelling or multi-sensor systems can be established.
- Some ISEs (like lead-ISE) operate in a small process range (e.g., working range pH = 4.0–6.9) [63]. Often, the ion-selective membrane (ISM) of the ISE destabilizes in low or high pH or salinity, hence their performance may be restricted.
- Potential drift in open-circuit measurements necessitates frequent calibration and long conditioning times prior to operation—sometimes even daily or more often [59]. ISEs should be used according to the maximum operating time, hence long-term usage beyond operating time can be critical.
- The ionophore-based polymer membranes (often PVC) are highly susceptible to mechanical stress and fouling/biofouling in complex media, which limits their performance in continuous applications [58]. Furthermore, plasticizers are prone to leaching, affecting aquatic lifeforms but also the stability and maintenance frequency of ISEs.
- Finally, the sensitivity of conventional potentiometric ISEs is limited by the Nernst equation (~59.2 mV per decade for monovalent ions), making it difficult to detect narrow concentration ranges (e.g., Na^+^ in blood: 135–145 mmol/L) or very small variations (e.g., ocean acidification: −0.002 pH units per year) with sufficient reliability [64]. Further preparation of the solutions or non-Nernstian approaches can help detect these narrow changes.

Despite these drawbacks, ISEs remain among the most versatile and practical tools for ion detection—and one of their most recognized challenges continues to be fouling.

## 5. Fouling Mechanisms on Ion-Selective Electrodes

Fouling describes the undesired deposition of molecules like proteins, lipids, and microorganisms on the electrode surface (or in case of ISE also on the ion-selective membrane), leading to drifts in signals, prolonged response times, lower sensitivity, and shorter lifespan of the sensor [60,61]. Further, reproducibility and limit of detection can be affected by fouling [62].

Determining an exact fouling degree, fouling rate, or fouling layer formation rate is usually difficult due to the complexity of the media composition (colloidal deposits and biofouling). Factors that can affect the extent of fouling include the composition, temperature, and flow properties of the medium; pH value; the (membrane) material; and the design of sensors and thus surfaces like membranes of sensors that are in contact with the medium. Other factors, such as geographical position, salinity, light, silt, competition between organisms, and others, can have a strong influence on fouling rates in specific applications [59]. To evade fouling, a trend towards single-use sensors is rising [62].

In this review paper, fouling is categorized into three different groups: organic fouling, the deposition of organic molecules (e.g., proteins, polysaccharides, lipids, other organic matter); inorganic fouling, the precipitation or crystallization of salts, hydroxides, or mineral deposits; and biological fouling (biofouling), the attachment and proliferation of microorganisms, extracellular polymeric substances, and macrofouling.

In aquaculture systems, particularly in RAS, these fouling mechanisms are of major practical relevance. The continuous monitoring of parameters such as pH, dissolved oxygen, ammonium, or nitrate exposes ISEs to nutrient-rich and biologically active environments, which strongly promote both organic and biofouling. Such processes can cause sensor drift and reduced accuracy, directly influencing the reliability of water quality management and, consequently, the health and productivity of cultured species.

Whilst many works have documented fouling on ISEs regarding a qualitative approach, few works look at quantitative fouling kinetics, such as fouling deposition rates in correlation to environmental parameters. Cecchoni et al. state a lag phase of 1 week without appreciable effects of NH_4_^+^ ISE in activated sludge processes and suggest cleaning routines of 1 week; ongoing usage leads to an exponential increase in response time of the sensor, and after approximately 1 month a plateau in fouling is reached [65]. Over time, a change from reversible fouling (2–4 weeks) to irreversible fouling (3–5 months) occurs, and response times remain slow and show the ageing of the electrode even after cleaning [65]. Furthermore, Grzegorczyk et al. (2018) suggest a Gompertz-sigmoidal-function-like biofilm growth kinetics, with a short initial running-in phase and exponential growth followed by a saturation phase, providing examples for lag phases (λ = 2.8 ± 0.2 d for initial extracellular polymeric substances and λ = 6.4 ± 0.4 d for late extracellular polymeric substances, respectively) and specific growth rates (0.27 ± 0.009 h^−1^ for biochemical preconditioning, 2.80 ± 0.2 d^−1^ for initial extracellular polymeric substances, and 6.4 ± 0.009 d^−1^ for late extracellular polymeric substances, respectively) [66]. Lyu et al. (2020) state drifts for solid-contact ISEs from 0.0117 mV/h to 1.4 mV/h [67], which depend strongly on both membrane formulation and environmental conditions. Furthermore, membrane-blocking is notable via drifting of ISEs in wastewaters after 20–48 h.

### 5.1. Biofouling Processes and Formation Mechanisms

**Figure 3:**
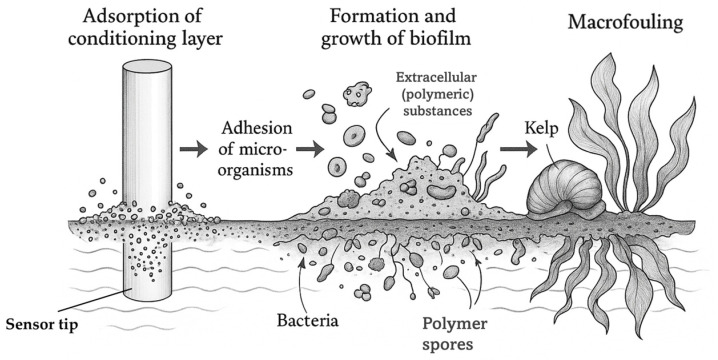
Schematic representation of biofouling processes and formation mechanisms on an ion-selective electrode sensor tip.

Biofouling (analogous to fouling) is defined as the unwanted accumulation of organisms (and biofilms) on exposed, submerged, or partially submerged sensor surfaces [68,69]. Generally, the phenomenon manifests itself wherever a non-sterile medium encounters a surface and provides adapted, undesirable properties (technical, health-related, or environmental) [31]. Among other things, biofouling leads to reduced efficiency of plant components, increased risk of contamination from biofoulants, increased likelihood of corrosion, and an increase in the probability of failure of plant components [31,70]. Biofouling occurs more frequently in warm regions/seasons like the tropics, as temperature directly affects growth of fouling species (increasing in summer and decreasing in winter, respectively) [71]. It usually consists of four stages, as shown in Figure 3: adsorption of a conditioning layer (I.), adhesion of microorganisms (e.g., bacteria) (II.), formation and growth of a biofilm (III.), and macrofouling (IV.)

Figure 3 illustrates the continuous development of biofouling, from the adsorption of an initial conditioning layer to bacterial adhesion, biofilm development, and macrofouling. Within a short time (1 min–1 h [72]), proteins and carbohydrates adsorb to a submerged surface (e.g., stainless steel [73]) in an aqueous environment. In this process, no continuous film is formed; instead, strongly varying, heterogeneous sections with different thicknesses develop [71]. This can lead to a slight increase in membrane resistance regarding ISEs. Next (1 h–1 d [72]) bacteria attach to the surface and form colonies which secrete extracellular (polymeric) substances (mainly proteins and polysaccharides, e.g., hexoses [71]) and a biofilm forms on the surface (1 d–1 w [72]), which induces a measurable drift for ion-selective electrodes. The biofilm is heterogeneous, varies according to the composition of the bacterial strains/microorganisms, and traps other isolated organisms, such as spores of algae or fungi [71], leading to a delayed response time of electrodes. Finally (2 w–1 m [72]), larger marine organisms, such as mussels or kelp, settle on the surface. This phenomenon is called macrofouling and takes place over a period of days to weeks [71] and can lead to partial or complete loss of ISE signals.

Biofouling is a complex process that can lead to the formation of widely varying biofilms depending on the prevailing conditions, such as the properties of the medium, including pH and organic composition in the form of lipids, proteins, and polysaccharides; the presence of bacteria, other microorganisms; and their metabolites, and multicellular species or large marine organisms [74,75]. Furthermore, the choice of surface hardness influences biofouling development: most aquatic fouling organisms tend to prefer hard surfaces, with approximately 127,000 identified species on hard surfaces and 30,000 on soft surfaces, respectively [76]. In addition, factors such as wettability (hydrophilic, hydrophobic, superhydrophilic, and superhydrophobic), colour, and micro texture also affect biofilm composition [76]. Moreover, biofouling communities vary temporally (due to seasonality in populations, arrival of new recruits, and growth periods) and spatially on a small and large scale (due to planktonic events, positional choices of attachment and settlement, mortality, and environmental influences) [77].

In aquaculture systems, biofouling is the dominant fouling type. Sensors used in fish tanks or RAS loops are continuously exposed to microbial populations, feed residues, and metabolic by-products. Biofilm formation on ISE membranes can rapidly lead to signal drift, delayed response times, or complete sensor failure. The high organic load and nutrient availability in aquaculture water further accelerate microbial attachment and biofilm maturation, making biofouling control a key challenge for reliable water quality monitoring.

### 5.2. Biofilm Development and Structural Characteristics

Some organisms are able to perform biofouling without an existing biofilm (e.g., barnacles of the genus *Amphibalanus amphitrite*) [75]. If a biofilm is formed, it appears as a film that contains algae, bacteria and/or fungi, and organic and inorganic substances, separating them from the surrounding environment. Generally, a biofilm is formed in four stages: 1. Attachment, 2. Proliferation, 3. Maturation, and 4. Dispersion [78]. The properties and structure of the biofilm vary greatly due to different biofilm organisms, physical conditions (including hydrodynamics, temperature, and surface properties), and chemical conditions (inhibiting substances, nutrient levels, and surface composition) [79]. Other factors that influence biofilm formation include pH, EC, DO concentration, region, season, light, and thereby depth of a water body [80]. A biofilm can significantly increase the tolerance of organisms to stressful environmental influences, which reduces the effectiveness of cleaning strategies [74].

Biofilms in aquaculture environments develop not only on sensors but also on tank walls, pipelines, and filtration units, where they affect both water quality and measurement accuracy. Once formed on ISE membranes, they can entrap nutrients and metabolites, locally altering ionic strength and pH. Such changes may result in sensor misreadings that compromise automated control processes such as aeration, feeding, and water recirculation in RAS.

### 5.3. Fouling Effects on Ion-Selective Electrode Membranes

Ion-selective electrodes often use a polymeric semi-permeable membrane, which is ion-selective, hence permeable only to a specific kind of ion. A correlation between fouling in membrane technology, especially regarding (ion-exchange) polymeric membranes [81], and fouling on ion-selective electrodes (here a PTFE membrane) [65] can be established.

According to Qi et al. (2022), fouling on ion-selective electrodes arises from nonspecific adsorption of substances onto the membrane [4]. Polymeric ISEs mostly suffer fouling of lipophilic sources (e.g., proteins, lipids, microorganisms, and oil) due to their liquid-like hydrophobic surface. Selectivity, stability, and lifetime of sensors can be influenced by fouling [4]. General influences of different fouling types according to Qi et al. (2022) are represented in Table 3 [4].

On one hand, sensors can be completely inactivated, e.g., by blocking semi-permeable membranes [82]. On the other hand, fouling (biofilm growth) can be limited to a maximum degree or level (here, a total population of 10^5^/mm^2^ was noted as the maximum limit; further exposure to fouling media does not affect the fouling layer/resistance) [83]. In aquaculture environments, such fouling-induced sensor inactivation directly affects operational control. Faulty readings of pH, ammonium, or nitrate can lead to suboptimal oxygenation, feed dosing, or recirculation control. Therefore, understanding fouling effects on ISE membranes is essential for maintaining accurate and continuous real-time monitoring in RAS and similar systems.

### 5.4. Impact of Fouling and Biofouling on Sensor Performance

Biofouling, in combination with inorganic or organic fouling, significantly affects the response time of pH sensors [65,79]. Diffusion limitations to and from the membrane, as well as blockage of the electrode’s liquid junction, can occur. Fouling layers increase the effective membrane thickness, thereby extending the diffusion path for ions to reach the electrode surface. At the same time, they raise the membrane resistance, analogous to the *resistance-in-series* model known from membrane technology, resulting in a prolonged sensor response time [79]. Moreover, the buffering capacity of biofilms and their secreted substances can further influence the sensor’s reaction kinetics. Biofilms also alter the local pH value at the electrode surface, reducing measurement accuracy and sensitivity, and may even distort sensor readings [31,79]. Interestingly, visible biofouling on sensors has been reported predominantly at depths lower than 75 m [84].

Fouling of electrodes in electrochemical analyses can affect their analytical characteristics, including sensitivity, detection limit, reproducibility, and reliability, thereby influencing the overall process performance [85]. Electrode fouling refers to the passivation of the electrode surface by adsorbed foulants, forming a progressively impermeable layer that prevents analytes from reaching the electrode surface [85].

Fouling typically occurs at specific surface features, such as edges or sites with favourable physicochemical properties, and can be driven by hydrophilic, hydrophobic, and/or electrostatic interactions, depending on the chemistry of both the foulant and the electrode [85]. Hydrophilic fouling tends to be more reversible, whereas hydrophobic fouling is often more persistent, as hydrophobic interactions are entropically favourable in aqueous environments due to the exclusion of water molecules, a phenomenon known as the hydrophobic effect [85].

Potentiometric ion-selective electrodes are generally unaffected by changes in electrode surface area, but their potential stability may still be influenced by surface processes such as biofouling [86]. Therefore, the detection of fouling is essential for the effective regeneration or cleaning of ISEs, as well as for the development of preventive antifouling strategies.

**Figure 4:**
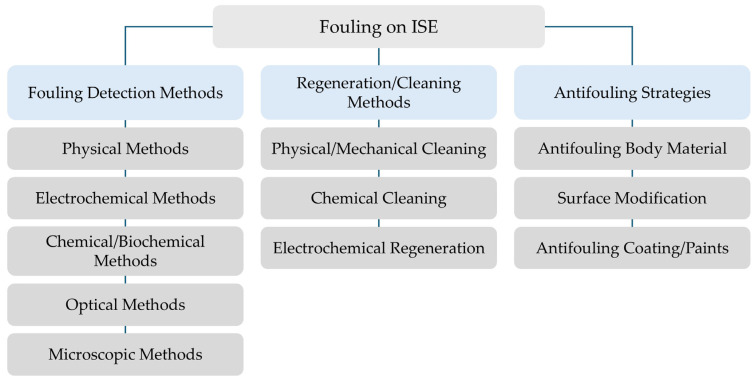
Schematics of fouling on ISEs covering detection methods, regeneration/cleaning approaches, and prevention strategies.

Figure 4 shows the operational logic to manage fouling on ion-selective electrodes. First, fouling detection is needed and performed by means of physical, electrochemical, and chemical/biochemical methods. After determining the fouling degree, appropriate regeneration/cleaning methods, such as physical/mechanical, chemical, and electrochemical cleaning/regeneration, are applied. To further ensure long-term stability of the ISE, preventive antifouling strategies (e.g., antifouling coatings, surface modifications, membrane engineering) are implemented.

### 5.5. Implementation of ISEs in RAS

The practical implementation of ISEs in RAS derives from three key performance indicators: (i) long-term drift, (ii) mean time between cleaning events, and (iii) the percentage of out-of-range readings.

Long-term drifting is a crucial parameter regarding long-term deployment of ISEs. Dissolved oxygen sensors and pH sensors drift approximately 2–5% over 6–12 months despite regular calibration; conventional ammonia ISEs exhibit a 3% drift over six months [87]. Yet, these values differ due to variables like membrane formulation and environmental conditions. Further usage of ISEs can lead to more frequent calibration to secure “accurate” measurements [87]. Operational longevity of ISEs stated by producers range from several months up to a year, depending on calibration frequency and medium composition. The mean time between calibration varies from daily (e.g., pH ISE Mettler Toledo InPro) to bi-weekly (NH_3_ ISE from Orion™) [87]. The percentage of out-of-range readings are not systematically reported in scientific studies.

A cleaning procedure should be implemented if the sensor’s slope varies by 10% of the initial slope or drifts by 5 mV, while recalibration could be used in the case of a slope deviation of 5% or an offset deviation of 5 mV. Furthermore, a sensor health score combining slope fidelity, drift stability, baseline offset, and response time performance, as well as impedance integrity, noise level, and redundancy consistency can help compare the health of sensors. Replacement may be required once the sensor’s slope differs by 15–20%.

In general, the hydrogen bonding and hydrophobic interactions with proteins, surface-blocking, and lipophilicity of the ISM determine whether the ISM is subjected to fouling by proteins, polysaccharides, or lipids, respectively [5]. Long-term drift and cleaning frequency change regarding these parameters.

Within aquaculture monitoring systems, such as RAS, these effects manifest as gradual sensor drift and delayed signal response, complicating automated feedback loops controlling aeration or dosing. Continuous exposure to organic waste, biofilms, and microbial metabolites further accelerates these fouling mechanisms. Effective fouling management on ISEs is therefore critical for ensuring stable, long-term, and accurate monitoring of water quality in aquaculture operations. To implement ISEs in RAS, operational thresholds or control indicators should be used to determine calibration, cleaning, and replacement frequency. Such indicators could be the critical comparison of fouling rates and their influence on performance, Nernst slope deviation, baseline/offset drift, response time, potential drift (reference electrode stability), leakage/internal electrolyte level, and slope consistency across multi-point calibration.

## 6. Detection and Monitoring Methods for Fouling on Ion-Selective Electrodes

The detection and mitigation of fouling on sensors—particularly ISEs—are crucial for ensuring long-term measurement stability and accuracy. Various methods exist to identify different types of fouling phenomena and to assess their extent and severity, but only a few are for sensor surfaces. Fouling detection depends strongly on the process environment and surrounding operational parameters. For instance, biofouling can be detected by monitoring the conductivity of the medium via conductometry, since the conductivity of culture media correlates with microbial growth and fermentation activity [88].

In aquaculture monitoring systems, early detection of fouling on ISEs is vital for maintaining stable real-time measurements of key parameters such as pH, dissolved oxygen, or ammonium. Biofilm formation on sensor surfaces can cause drift or delayed response, leading to misinterpretation of water quality data and consequently to inefficient control of aeration or feeding regimes. Reliable fouling detection techniques therefore contribute directly to the health management and productivity of aquaculture systems, particularly RAS.

Common fouling detection techniques for sensors, including ISEs, comprise the following (see Figure 4):

- Physical methods such as quartz crystal microbalance (QCM) [50] and surface plasmon resonance (SPR);
- Electrochemical methods such as potentiometry and electrochemical impedance spectroscopy (EIS) [50];
- Chemical and biochemical methods including staining with dyes or radioactive labelling [86];
- Optical techniques such as UV/VIS and fluorescence microscopy; and
- Microscopic techniques such as scanning electron microscopy (SEM) [50] and atomic force microscopy (AFM);

Blaen et al. (2016) additionally reported reflected light microscopy and X-ray diffraction (XRD) for characterizing the membrane surface of ISEs [26]. A comparison of sensitivity, reproducibility, and suitability of different detection methods is listed in Table 4.

Chemical cleaning followed by analysis of removed foulants can also provide quantitative and qualitative insights into the degree of fouling. Importantly, even partial surface coverage by fouling can significantly impair electrode performance [26]

### 6.1. Physical and Optical Methods for Fouling Detection

In many cases, fouling effects can be observed visually [89]. Colorimetric ion-selective sensors, for example, exhibit colour shifts due to changes in chromophores when the target analyte also acts as a foulant. Hence, a sensor immersed in a standard solution should display a characteristic colour change [90].

Chemical staining techniques employing dyes such as Carnoy’s solution, toluidine blue, or SYTO 9 are used to visualize bacterial growth and biofouling on sensor surfaces [89,91]. Subsequent analysis via (confocal) laser scanning microscopy enables high-resolution detection of stained biofilm structures [92]. Other optical methods, including UV/VIS spectroscopy and image analysis, are also applied, although results should be interpreted cautiously, as they may not linearly correlate with fouling severity [83].

Raman spectroscopy can detect fouling on both amperometric and potentiometric ISEs [93], while field emission scanning electron microscopy (FESEM) provides detailed surface characterization [94]. X-ray photoelectron spectroscopy (XPS), also known as electron spectroscopy for chemical analysis (ESCA), can determine membrane composition and chemical binding states [26].

The quartz crystal microbalance (QCM) is another valuable method for fouling quantification. It measures mass changes on the sensor surface by comparing the resonance frequency before and after exposure [95]. QCM can also be used for real-time monitoring of mass accumulation during fouling processes [96].

In aquaculture applications, such physical detection methods can reveal biofilm accumulation on submerged ISEs before critical signal drift occurs. For instance, QCM-based monitoring could be adapted for in situ evaluation of fouling rates in nutrient-rich aquaculture water, helping operators schedule cleaning or regeneration cycles before measurement failure affects stock management.

### 6.2. Electrochemical Methods for Fouling Detection

Indirect monitoring of parameters such as EC or pH can provide early indicators of fouling. A correlation between conductivity and biofouling growth has been established, as conductometric methods enable the detection of bacterial activity on sensors [97]. In harsh operational environments—such as under highly acidic or alkaline conditions—changes in pH, combined with exposure time, can be used to estimate an additional fouling rate. While low pH (<4) increases inorganic precipitation and membrane swelling, high pH (>10) increases hydroxide precipitation and the adsorption of proteins. High salinity leads to faster colonization by halophilic organisms and densification of extracellular polymeric substances; high organic load accelerates the formation of extracellular polymeric substances and biofilm thickening. These processes are strongly influenced by flow limitations (stagnant zones, increased attachment, and diffusion limitation) and temperature (accelerated microbial and biofilm growth).

Because ion-selective electrodes operate based on electrochemical reactions, a variety of electrochemical techniques can be employed for fouling assessment, including the following [98]:

- Chronopotentiometry,
- Chronoamperometry,
- Pulse amperometry,
- Differential pulse voltammetry,
- Conductometry, and
- Polarization measurements.

Using a combination of these techniques provides a more comprehensive and reliable evaluation of fouling on ISEs. Different sensor types (e.g., potentiometric or amperometric) require specific detection methods suited to their transduction mechanism.

Usually, sensors exhibit a nonlinear performance decline over time: initial fouling may have little effect, but once a critical fouling threshold is reached, a rapid and disproportionate decrease in sensitivity, stability, and lifespan occurs. Such behaviour can be visualized and monitored in real time [83].

In aquaculture water monitoring, electrochemical methods, particularly EIS and potentiometric analysis, are well suited for continuous observation of sensor stability in RAS loops. Detecting impedance changes or potential shifts in situ allows early recognition of biofilm growth and supports timely sensor maintenance, reducing the risk of inaccurate pH or ammonium readings that could endanger fish health. A comparison of different fouling detection methods for ISE is shown in Table 5.

### 6.3. Potentiometric Evaluation of Fouling Effects

The functionality of ISEs can be evaluated using potentiometric measurements by recording electrode responses at various standard concentrations [98]. Changes caused by fouling can be identified through variations in electrode slope, while electrode ageing can be indicated by a gradual increase in internal resistance. For example, the slope of a new electrode may decrease from 60 mV/decade in a standard solution to 40 mV/decade after extended use, indicating a measurable degree of fouling [99]. Temperature control is essential during such measurements, as a 10 K variation can approximately double the electrical resistance.

A straightforward method for fouling detection involves measuring the direct current (DC) resistance of the ion-selective membrane. Fouling correlates proportionally with increasing resistance until a critical threshold is reached, after which the resistance rises sharply. Typical membrane resistance values are 10–20 MΩ for membranes containing functional ionophores, 2–4 MΩ for those with ion-exchanger inclusion, and 300 MΩ for membranes without ionophores [100].

A promising in situ technique combines potentiometry with null ellipsometry, allowing real-time monitoring of biofouling via both the electrode potential and the corresponding ellipsometric signal, which reflects the amount of adsorbed protein (e.g., bovine serum albumin) [86]. Chronopotentiometry is another electrochemical method in which a controlled current is applied between two electrodes, and the potential of one electrode is measured over time relative to a reference electrode. Fouling of ISEs leads to changes in the bulk resistance of the electrode, making chronopotentiometry a useful tool for evaluating membrane resistance and fouling progression [101,102].

For aquaculture monitoring, potentiometric analysis is particularly relevant, as it directly evaluates sensor performance under real-water conditions. Continuous observation of electrode slopes and resistance trends can serve as diagnostic indicators for biofilm growth or salt deposition, both of which affect the accuracy of nutrient and oxygen monitoring in intensive aquaculture systems.

### 6.4. Amperometric Detection and Fouling Assessment

Amperometry is an electrochemical technique used to determine the concentration of redox-active species [103]. Pulsed amperometry, employing a periodic voltage waveform, enhances sensor robustness and can even serve as an electrochemical cleaning method for noble metal electrodes [104]. Fouling of Clark-type dissolved oxygen sensors can be assessed by measuring the oxygen transfer time across the membrane of the Clark cell. In this approach, one electrode operates continuously under steady-state conditions for oxygen detection, while another is energized intermittently to detect fouling effects. However, this method still requires reference calibration (lookup tables) to compensate for fouling-induced deviations [105].

Within aquaculture, amperometric approaches are essential for maintaining accurate dissolved oxygen readings. Since oxygen is a critical parameter in RAS operation, detecting membrane fouling through amperometric signals allows proactive cleaning or recalibration, ensuring stable oxygen supply for aquatic organisms.

### 6.5. Voltametric Techniques for Evaluating Electrode Fouling

Cyclic voltammetry (CV) is an electrochemical technique in which the potential of the working electrode is cyclically varied while the resulting current is recorded relative to a reference electrode [106]. This method has been widely used to determine fouling rates on various electrode materials, including carbon electrodes [107] and reference electrodes such as Ag/AgCl [108]. Applied to aquaculture sensors, cyclic voltammetry could serve as a diagnostic method to assess the degree of electrode passivation or biofilm formation on reusable sensor tips, supporting the design of maintenance schedules and surface treatment protocols for long-term use.

### 6.6. Electrical Impedance Spectroscopy (EIS) for Real-Time Fouling Monitoring

Electrical impedance spectroscopy (EIS) is a well-established technique for detecting and quantifying fouling. It has been applied to real-time monitoring of fouling in membrane processes [109] and can also be used to assess fouling on electrode surfaces, as fouling layers alter the magnitude and phase of the impedance response [110]. Fouling-induced changes in impedance provide direct insight into surface deposition phenomena within sensor housings [110]. EIS can also indicate biofilm formation by applying a sinusoidal voltage signal to the electrode and monitoring the temporal response; variations in impedance spectra correspond to microbial growth and biofilm development [97]. Additionally, EIS enables the determination of fouling layer thickness by quantifying the electrical resistance associated with the deposited film. Experimental results have shown that biofouling contributes to the overall charge transfer resistance of ion-selective electrodes, thereby reducing ion permeability and impairing signal stability [111].

In aquaculture environments, EIS is particularly promising for non-invasive, real-time evaluation of sensor integrity. Continuous impedance tracking could provide early warning of biofilm buildup on ISEs, allowing corrective actions before data drift impacts automated control of water quality and fish health.

### 6.7. Critical Comparison of Fouling Detection Methods

As mentioned, various methods can be used to detect fouling on surfaces, like ISE-type sensors, but only a few are suitable for aquacultures and even fewer work inline. Highly sensitive laboratory equipment (QCM, SPR, SEM, AFM) provide reproducible, highly valued information, but are difficult to operate and need specialized equipment and sample preparation, hence providing a rather laboratorial approach. Optical methods are partly practical for aquacultures; their insensitivity (UV/VIS, turbidity) can lead to falsified conclusions, while other methods (fluorescence microscopy) require expensive equipment and/or dyes. Electrochemical methods are quite suitable for real-time monitoring of fouling on ISEs and are field-proven, whilst EIS is very sensitive and detects biofilm growth; however, additional hardware is needed, as well as expertise in evaluation. Potentiometric fouling detection is quite simple and provides only general fouling information without distinguishing between different fouling types.

## 7. Regeneration and Cleaning Methods for Ion-Selective Electrodes

The long-term operation of ISEs often results in surface contamination through organic, inorganic, and biological deposits that impair sensor accuracy, response time, and stability. Cleaning and regeneration methods are therefore essential to restore the electrode’s functionality by removing or mitigating accumulated foulants and reestablishing baseline performance. These methods differ in mechanism and intensity—ranging from mechanical and chemical to electrochemical or acoustic cleaning approaches—and are selected based on the electrode type and the nature of fouling. In aquaculture applications, where sensors are continuously exposed to nutrient-rich and biologically active media, effective regeneration protocols are indispensable to maintain measurement reliability and ensure accurate monitoring of critical parameters such as pH, dissolved oxygen, and ammonium.

Hereby it is necessary to differentiate between reversible and irreversible sensor fouling. Reversible fouling refers to foulants which can easily be removed (e.g., with preconditioning and routine maintenance) like conditioning a CO_3_^2−^-ISE for 15 h in Na_2_CO_3_ after being fouled by sodium alginate to regain the initial slope response, while irreversible fouling (like slime or macrofouling) cannot be cleaned off easily and needs further cleaning methods, such as mechanical, chemical, physical (and ultrasonic), electrochemical, integrated, and advanced cleaning methods [31].

### 7.1. Electrode Preconditioning and Routine Maintenance

Sensor drift and electrode dissolution can be mitigated by conditioning the ion-selective electrode in the sample matrix before measurements [78]. Additionally, electrodes can be rinsed or equilibrated in a low-analyte concentration solution between measurements to restore the baseline potential [112]. Reversible foulants can be removed via this method. Frequent cleaning and recalibration are essential for ISEs used in long-term monitoring applications, as they maintain sensor stability and measurement accuracy [112].

In aquaculture systems, where sensors are continuously exposed to nutrient-rich and biologically active water, regular conditioning and recalibration are vital to sustain data reliability. Frequent cleaning between measurement cycles minimizes biofilm accumulation and stabilizes readings of key parameters such as pH and ammonium, ensuring accurate process control and safeguarding aquatic species in RAS.

### 7.2. Mechanical Cleaning Approaches

Mechanical cleaning—such as the use of wipers or brushes—is an effective method for removing biofouling without damaging sensitive sensor components. Such systems should be designed to be removable or replaceable for long-term deployment [31,71]. 

Once a critical fouling degree is reached, foulants tend to adhere irreversibly even outside the sample matrix. Therefore, crystalline ISEs (e.g., pH glass electrodes) often require mechanical polishing, whereas polymeric ISEs typically demand membrane replacement [112]. Water jet cleaning has also been established for removing superficial deposits from non-delicate sensor components [71].

In aquaculture applications, mechanical cleaning systems, such as automated wipers integrated into submerged sensor probes, can significantly reduce biofouling during continuous operation. The ability to remove accumulated biomass without disassembling sensors is particularly beneficial for RAS environments, where uninterrupted real-time monitoring of water quality is essential.

### 7.3. Chemical Cleaning and Regeneration

For solid-state ISEs, chemical cleaning agents can restore electrode performance.

- Ascorbic acid treatment of sulphate-contaminated membranes reestablishes electrode response.
- Perchloric acid (HClO_4_) effectively removes lead sulphate and oxide layers.
- Ethylenediaminetetraacetic acid (EDTA) dissolves lead sulphate and enhances lead oxide removal efficiency [26].

Since fouling composition varies depending on the operating environment, cleaning protocols must be specifically tailored to the predominant foulants. Routine physical or chemical cleaning, followed by post-cleaning performance testing, ensures regeneration effectiveness and reproducibility.

In aquaculture environments, chemical regeneration protocols must consider the complex composition of organic and biological foulants, including proteins, polysaccharides, and microbial biofilms. Customized cleaning procedures employing mild and biocompatible reagents, such as diluted EDTA solutions or organic acids, are essential to preserve sensor functionality while preventing the introduction of toxic residues into the aquatic system.

### 7.4. Physical and Ultrasonic Cleaning Techniques

Alternative cleaning techniques from other industries can be adapted for sensor regeneration. Ultrasonic irradiation has been demonstrated to control biofilm formation, enhance the efficiency of biocides, and facilitate biofilm detachment. Similarly, low-frequency acoustic vibration disrupts larvae attachment and prevents resettlement on sensor surfaces [38].

For aquaculture monitoring systems, physical cleaning methods such as ultrasound present promising non-invasive options for in situ maintenance. Ultrasonic waves can loosen biofilms and particulate fouling without requiring chemical exposure, reducing maintenance time and preventing contamination risks in live fish tanks.

### 7.5. Electrochemical Cleaning and In Situ Fouling Control

Electrochemical fouling control mitigates fouling by in situ generation of biocides near the electrode surface or by modifying electron transport through the fouling layer [38]. This method can function both as a preventive and regenerative strategy and has shown promise for solid-contact ISEs operating in complex aqueous environments.

In the context of aquaculture, electrochemical cleaning could be integrated into smart sensor platforms to periodically remove surface deposits through controlled potential pulses. This approach allows autonomous regeneration of electrodes and extends operational lifespan under biofouling-prone conditions characteristic of RAS water loops.

### 7.6. Integrated and Advanced Regeneration Strategies

Chemical cleaning agents such as chlorine, acids, and alkalis are widely used for general fouling removal. However, some of these agents (e.g., chlorination in cooling systems) can produce harmful by-products such as trihalomethanes [71]. Emerging environmentally friendly strategies include the use of bio-based antifouling agents, such as naturally secreted enzymes or extracellular compounds that inhibit the formation of fouling layers.

Because no single cleaning approach fully removes or prevents fouling, combined cleaning strategies, integrating physical, chemical, acoustic, and electrochemical methods, offer the most effective and sustainable long-term antifouling solutions for ion-selective electrodes.

Hybrid cleaning approaches are particularly advantageous for aquaculture systems, where combining gentle physical cleaning methods (e.g., wiping or ultrasound) with periodic electrochemical or enzymatic regeneration minimizes downtime and prevents biofilm stabilization. Sustainable, non-toxic cleaning routines are crucial for maintaining water quality and ensuring compliance with environmental safety standards in fish production environments.

### 7.7. Critical Comparison of Cleaning Methods

Cleaning methods for fouled ISEs differ widely in their practicability, effectiveness, and impact on the sensor. For long-term usage of ISEs, proper cleaning routines should be established. A list of compared cleaning methods is represented in Table 6.

Widely spread chemical cleaning is effective against inorganic and organic foulants, but may lead to harsh conditions damaging the sensor (e.g., plasticizer or ion exchanger of ISM). Mild chemicals can be sufficient for sensor cleaning whilst creating neglectable sensor changes, but maintenance frequency or duration may be higher.

The compatibility rating (“safe”, “medium”, “not safe”) refers to the chemical stability of conventional ISE membrane materials exposed to cleaning agents (Table 7).

The same problem may appear with mechanical cleaning, as wipers or polishing may cause harm to the membrane of the ISE. Enzymatic cleaning methods may be gentler, but are more expensive and tend to work in limited operational ranges with limited stability.

A promising approach is given by electrochemical cleaning methods, as they require no chemical reagents and can be integrated into ISE construction. This approach remains quite experimental, as there are not many sensors with built-in electrochemical cleaning methods.

For RAS, a cleaning method consisting chemical cleaning (conditioning or mild reagents) as well as gentle mechanical cleaning (wiping or polishing) should be considered. Future ISE manufacturers should integrate built-in self-cleaning strategies, like electrochemical cleaning.

## 8. Antifouling Strategies for Ion-Selective Electrodes

While regeneration methods focus on restoring sensor performance after fouling has occurred, preventive antifouling strategies aim to minimize or entirely prevent foulant accumulation on ISEs. Such measures are particularly valuable for long-term monitoring applications, as they extend sensor lifetime, reduce maintenance frequency, and ensure consistent data quality in complex aquatic environments, though they differ in durability and response time effects.

In RAS, where sensors operate continuously under nutrient-rich and biologically active conditions, preventive strategies are essential to maintain stable monitoring of key parameters such as pH, ammonium, and dissolved oxygen.

Preventive antifouling concepts have been widely developed and implemented across various industrial sectors, including membrane technology [113], heat exchangers [114], and sensor systems [4,31,71,89,115,116,117,118,119,120]. Although many biocidal antifoulants are highly effective, their environmental and health impacts, particularly their non-selective toxicity [121], have driven a shift toward sustainable, environmentally compatible alternatives [122].

### 8.1. Fundamentals and Mechanisms of Fouling Prevention

Fouling is primarily driven by the physicochemical characteristics of sensor surfaces, including surface roughness, hydrophilicity, surface charge, and surface free energy [4]. Furthermore, the presence of a hydration layer, the ionic strength and pH of a medium, and the exposure towards the medium and light (e.g., UV) influence fouling characteristics. Thus, antifouling effectiveness depends heavily on the choice of sensor materials, surface modification, and coating technologies. Preventive measures also include sample pretreatment, controlled process environments, and optimized calibration routines [4].

In aquaculture monitoring, where high biological activity promotes biofilm formation, controlling these parameters directly influences sensor reliability and reduces the need for frequent recalibration. Coatings or thin films are the most frequently described antifouling methods in the literature [85]. Nature-inspired approaches, such as limiting light exposure to inhibit algal growth, can provide simple, passive antifouling solutions. In practical applications, reducing exposure time (e.g., immersing sensors only during measurement) can further minimize fouling on ISEs [71]. Antifouling systems can generally be classified as biocidal, biomimetic, micro-textured, superhydrophobic, hydrophilic, amphiphilic, hybrid, or active cleaning systems [123].

### 8.2. Selection of Antifouling Construction Materials for ISEs

The selection of materials for ISE construction determines both mechanical durability and fouling resistance. Common sensor bodies include glass electrodes for standard pH or Na^+^ measurements; bronze or antimony electrodes for industrial applications [124]; enamelled steel probes for extreme conditions [125]; and optical or hydrogel-based pH probes for specialized systems [126,127].

In aquaculture systems, where sensors are exposed to high salinity variations and organic loading, robust materials such as enamelled steel or carbon composites are particularly advantageous for long-term stability.

Fouling typically results from adsorption of organic molecules or biological cells, leading to signal deterioration and reduced performance [4]. Nanoporous carbon electrodes show superior fouling resistance because large foulants are sterically hindered from entering internal pores [85].

Recent developments focus on carbon-based materials, such as graphene, diamond, and carbon composites, which exhibit high chemical inertness, low protein and lipid affinity, easy surface functionalization, and electrocatalytic activity. These materials are well suited for long-term and in situ measurements [85]. Diamond, e.g., excels in fast response time due to high conductivity, whilst carbon composites show a moderate drift and good biofouling resistance.

Enamelled steel sensors demonstrate outstanding sensitivity and longevity by forming a self-renewing leaching layer that regenerates every one to two weeks in acidic or neutral media. Depending on layer thickness, lifetimes can exceed 100 years, while showing high resistance to solvents, oils, and temperature shocks [125,128]. Thus, enamelled steel excels in durability with a negligible influence on response time. Further promising sensor materials include diamond [129,130,131,132], antimony [133,134], and stainless steel alloys [135,136,137,138]. A list of possible materials for ISE construction is listed in Table 8.

### 8.3. Surface Modification Strategies for ISEs

A promising route to mitigate fouling is surface modification of the ion-selective membrane. Key antifouling characteristics, such as protein resistance, hydrophilicity, zwitterionic behaviour, and biocompatibility, are achieved by tailoring the surface chemistry and topography [116]. The combination of physical (e.g., roughness, charge) and chemical (e.g., coatings, grafted polymers) modifications can create synergistic antifouling effects, preventing microbial settlement and facilitating biofilm detachment [139]. In RAS environments, such surface-modified ISEs can maintain accuracy over longer intervals, reducing sensor replacement frequency and improving operational efficiency.

#### 8.3.1. Physical Surface Modification

Antifouling can be achieved by modifying exposed surfaces to reduce surface energy, introduce repulsive interactions with foulants, or control micro texture [140]. Surface roughness plays a decisive role: smoother surfaces generally reduce bacterial adhesion and biofilm formation, while increased roughness may enhance fouling and affect ion adsorption, selectivity, and membrane charge [111,141]. Surface roughness can be tuned by incorporating additives such as 5% PTFE into polymeric ion-selective membranes [111], which is suitable for production and use in aquaculture.

Biomimetic approaches inspired by marine organisms, such as shark placoid scales, pilot whale skin pores, or dolphin skin elasticity, provide antifouling functionality through micro- and nanostructures, but remain mostly experimental [71,142]. Other natural analogues include fish scales, crab shells, butterfly wings, and tree frog toe pads, which exhibit self-cleaning or anti-adhesive properties [142]. Additionally, modifying the surface charge toward more negative values reduces bacterial adhesion [111], while low-surface-energy coatings hinder microbial bonding and biofilm development [143]. These micro-structured and low-energy surface modifications are especially beneficial for sensors submerged in aquaculture tanks, where bacterial attachment rates are high.

Photocatalytic self-cleaning coatings based on anatase TiO_2_ exhibit strong antimicrobial and anti-organic fouling properties. Under UV illumination, reactive oxygen species (ROS) are generated, degrading organic contaminants into water and carbon dioxide [76]. These coatings also form superhydrophilic films, further inhibiting organic adhesion and enhancing antifouling stability on ISE surfaces, but remain semi-practical due to the necessity of UV light [92].

#### 8.3.2. Chemical Surface Modification

Polyethylene glycol (PEG) remains a widely used surface modifier due to its low cost and ready availability. However, zwitterionic coatings have recently gained attention for their superior antifouling performance, offering higher oxidation stability, lower immunogenicity, and improved biocompatibility compared to PEG [116]. Further chemical modifications include metallic nanoparticles, catalytic redox couples, nanoporous electrodes, and polymeric or non-polymeric films, all adaptable for ISE applications [85]. E.g., for biofouling prevention, ISEs coated with copper- and zinc-based layers achieved an average 59% reduction in biofilm formation [84].

Such modifications not only enhance antifouling resistance but also improve electrical stability and ion selectivity of the electrode interface. In aquaculture sensor systems, these coatings have proven to help maintain calibration stability even in biologically active water. To counter oil fouling on ISEs, zwitterionic polymer-based self-cleaning coatings have been developed and successfully applied to calcium ISEs, demonstrating durable antifouling performance [89].

### 8.4. Antifouling Coatings and Functional Films for ISEs

**Figure 5:**
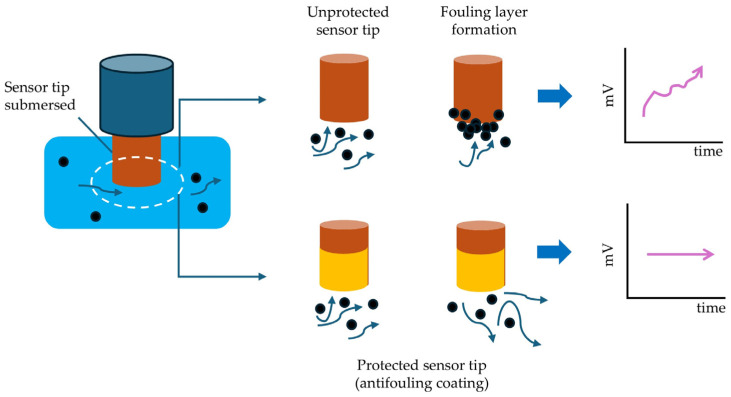
Schematic representation of fouling (drifting) and antifouling (no drift) phenomena on the measuring tip of an ion-selective electrode (ISE), showing protected (yellow, repellent) vs. unprotected (orange, not repellent) surfaces, according to [31].

Antifouling coatings serve as physical and chemical barriers between the electrode surface and the surrounding medium, as seen in Figure 5.

According to Dafforn et al. (2011), four main coating types are used in marine fouling control: (i.) soluble-matrix coatings, (ii.) contact-leaching coatings, (iii.) self-polishing copolymers, and (iv.) foul-release coatings [69].

For ISEs, current developments include non-stick coatings [38], biocide-impregnated layers [71], magnetic coatings [144], self-sterilizing coatings [145], and maintenance-free, self-polishing polymeric membranes [146]. These coatings minimize direct contact between the sensor surface and foulants. For aquaculture-monitoring probes, such coatings are essential to prevent rapid fouling in nutrient-rich, bioactive waters and to minimize maintenance intervals. However, many traditional antifouling paints release toxic substances into aquatic environments. One historically significant compound is tributyltin (TBT), once the most effective antifouling agent used in marine paints. Due to its extreme toxicity, bioaccumulation, and severe ecological impacts, TBT was banned globally under the IMO “International Convention on the Control of Harmful Anti-Fouling Systems on Ships”. This prohibition marked a fundamental shift toward tin-free and environmentally benign coatings.

Modern antifouling coatings now rely on silicone-based foul-release systems, fluoropolymers, and hydrophilic or zwitterionic polymer films. Biocide-free and naturally derived materials, such as chitosan, cellulose derivatives, and marine-organism extracts, are increasingly used to provide long-term stability with minimal environmental impact [69,147]. Although some coatings still require periodic renewal, embedded additives (e.g., biocides, fungicides, herbicides) continue to support active fouling prevention when applied in safe concentrations.

### 8.5. Emerging and Bioinspired Antifouling Concepts

Bioinspired antifouling strategies draw from natural defence mechanisms that prevent surface colonization in marine organisms.

- Hydration-layer formation prevents protein adsorption and microbial attachment.
- Micro- and nanostructured surfaces derived from sharks, molluscs, and lotus leaves minimize effective contact areas for biofilms.
- Bioengineered polymers incorporating natural antifouling moieties, such as zwitterions, amino acids, or peptides, represent a sustainable route for future sensor designs.

Hybrid systems combining physical texturing with chemical functionality often achieve synergistic effects, enhancing fouling resistance, self-cleaning ability, and sensor longevity under real operating conditions. Such bioinspired coatings could be directly applied to long-term aquaculture probes, improving monitoring accuracy and reducing manual maintenance. Whilst only superhydrophilic and hydrophilic zwitterionic coatings for use in aquaculture exist, micro-structured surfaces, self-cleaning surfaces, and fish-scale/shark-skin patterns fail to be implemented in aquacultures due to the difficulty of mass production for electrodes, experimental and pre-commercial conditions.

### 8.6. Environmentally Friendly Antifoulants and Emerging Coating Materials

Growing awareness of the environmental impact of toxic antifouling agents has accelerated research into non-toxic and naturally derived alternatives, particularly in the maritime sector. Modern foul-release coatings frequently employ silicone elastomers, waxes, or oils, while bio-based antifoulants are often derived from marine organisms, such as algae [69]. Typical foul-release materials used in marine applications include silicones, fluoropolymers, and silicone hydrogels [69]. For aquaculture sensors, these environmentally benign coatings are especially valuable to avoid toxic leaching into fish tanks.

Recent studies also explore specialized hydrogels and bacteria-producing extracellular antifouling components as potential alternatives. However, these approaches face challenges such as inconsistent antifouling performance and the complex maintenance of living antifouling systems [71]. The range of antifoulants reported in the literature, including natural compounds, foul-release materials, polymeric and photocatalytic coatings, and metallic or zwitterionic systems, is summarized in Table 9.

Because some organisms (e.g., certain algae) tolerate copper-based antifoulants, booster biocides are often incorporated into paints to enhance efficacy. These coatings are preferred in RAS as they do not release toxic metals, have no pH-dependent toxicity, do not interfere with sensor signals, and in general do not leach toxins that pose mortality risks to the cultivated organism, while also meeting sustainability requirements and thus regulatory pressure.

### 8.7. Ionic Liquids

To prevent leaching and drifting, multifunctional additives called ionic liquids are used. These liquids are used to exchange conventional lipophilic salts (e.g., KTFPB, DOS, NPOE), which results in less leaching/washing out, a stabilized membrane matrix with less pores and hence less biofilm adhesion, and smoother homogenous surfaces preventing adsorption of proteins and extracellular polymeric substances. Ionic liquids work as ion exchangers, transducers and plasticizers simultaneously, providing a stabilized Nernst slope, less drifting, and less membrane ageing.

Morawska et al. (2024) used the ionic liquid 1-hexyl-3-methylimidazolium instead of using conventional lipophilic additives, resulting in less membrane leaching and hence lower degradation of the ISM [63]. The resulting membrane showed improved stability, less drifting, and a prolonged longevity compared to conventional membranes. Due to its multifunctionality, trihexyltetradecylphosphonium chloride works as an ion exchanger, lipophilic ionic component, and membrane-phase transducer, reducing membrane resistance and stabilizing the potential of internal electrodes which results in a more robust membrane with less alteration. Deployed daily over 4 months, the sensor shows minimal variation in Nernst response compared to a fresh sensor (−58.9 mV/decade and −60.1 mV/decade, respectively) [148]. A comparison of ionic liquids and conventional additives as an antifouling method is listed in Table 10.

### 8.8. Critical Comparison of Antifouling Methods

As nanoporous carbon and diamond excel in mechanical and chemical inertness and hence low foulant affinity, they are suitable for SC-ISE but require specialized manufacturing. Furthermore, enamelled steel sensors show promising long-term usage. For conventional (e.g., polymeric) ISEs, further modifications are needed.

Physical surface modifications show significant antifouling properties, but remain mostly experimental due to being difficult to integrate in production and their proneness to damage through abrasion. The most common and suitable surface modification method is the chemical method via easy-to-apply coatings made of zwitterionic, hydrophilic polymers, showing strong antifouling properties with low sensor performance interference. Despite these advantages, they suffer from low long-term durability, especially in harsh environments (high salinity).

Antifouling coatings have been proven to differ strongly in toxicity, and hence also in environmental safety, effectiveness, cost, influence on sensor performance, and durability. Due to the famous history of TBT, biocide-based coatings have proven to be unsuitable for aquacultures. Despite their effectiveness and low sensor impact, regulatory restrictions, as well as their toxicity and variable durability (leaching) remain challenging. TiO_2_ photocatalytic coatings have been proven to prevent microbial adhesion as well as degrade organic contaminants, but due to their dependency on UV light, they are limited in their applications. Preventing protein and EPS adsorption, hydrophilic and zwitterionic coatings outperform PEG in antifouling properties and stability, but remain mostly experimental. Best suited for aquacultures are silicone foul-release and fluoropolymer-based coatings due to their high durability and low leaching. Their main disadvantage lies in altering the sensors performance (e.g., response time). A general Comparison of different antifouling coating types can be found in Table 11. 

Overall, ionic liquids exceed conventional additives in antifouling mechanisms by reducing membrane leaching and membrane components, stabilizing the Nernst response, and extending operational periods, hence providing promising candidates for real-time monitoring in aquacultures. Their disadvantages are mostly their high cost, experimental approach, and possible, not-yet documented interactions with harsh media.

Because fouling is a multifactorial process, no single antifouling strategy is universally effective. Combined preventive approaches, integrating optimized sensor materials, advanced surface modifications, and dynamic operational controls (e.g., intermittent cleaning cycles or in situ electrochemical regeneration) provide the most robust, long-term solutions. In recirculating aquaculture systems, such integrated strategies enable continuous, low-maintenance operation and stable control of critical parameters such as oxygen and ammonium.

## 9. Future Perspectives

Future research should focus on the following:

- Developing sustainable, non-toxic antifouling materials: Future approaches should focus on coatings with negligible ecotoxicity, even in high salinity or other harsh environments.
- Exploring adaptive or self-healing coatings that respond to environmental stimuli: Integrating self-cleaning and self-regenerating functions into smart ISE systems for autonomous, long-term water quality monitoring. This can be achieved via photocatalytic layers, wiper systems, or electrochemical cleaning pulses, but needs to be validated in realistic settings.
- Development of ionic liquids for antifouling membrane formulations: It has been shown that ionic liquids play a promising part in reducing leaching and potential drifting for ISM. Further investigation of different ionic liquids in realistic aquaculture settings should be tested to determine long-term stability and membrane–media interactions.
- Combination of different antifouling techniques to improve effectiveness: Carbon-like and steel-based electrode bodies excel in antifouling properties and long-term stability. Combined with chemical modification, further features like continuous use in RAS can be altered.
- Field vs. laboratory validation: Most antifouling approaches still remain untested in field approaches and are only validated under controlled laboratory environments. Future approaches should validate and compare antifouling techniques in realistic aquaculture-based environments.
- Self-monitored ISE with health diagnostics: Integrated, inline fouling analysis of ISEs can help with drift compensation and response time monitoring, and index a sensor health score, which can be used to reduce manual maintenance.
- Standardization of operational thresholds and performance limitations: To facilitate comparison between antifouling methods, a standardized routine protocol regarding the usage of sensors in aquacultures (e.g., drift limits, calibration interval) as well as performance limitations (e.g., replacement criteria) should be implemented.

## 10. Discussion

Real-time monitoring plays a crucial role in industrial and environmental applications, as it provides immediate feedback on critical parameters and enables predictive control of complex processes. Sensors are the cornerstone of such systems, delivering continuous information on process dynamics and environmental conditions, including those in aquaculture. However, during long-term deployment, sensors are highly susceptible to fouling and biofouling, particularly in aqueous environments. This susceptibility leads to signal drift, data degradation, increased maintenance demand, and higher operational costs.

In recirculating aquaculture systems, these effects are particularly critical: sensor fouling can directly compromise oxygen control, pH balance, and ammonium regulation, parameters that are essential for fish health and production efficiency. Continuous operation in nutrient-rich, biologically active water accelerates fouling and biofilm formation, making the prevention and timely mitigation of these effects indispensable for reliable sensor performance.

Among the most important sensors used in aquatic monitoring are ISEs, which measure key parameters such as pH and specific ion concentrations. ISEs operate in potentiometric, amperometric, or voltametric modes, with selectivity ensured by ion-selective membranes incorporating specific ionophores. Owing to their sensitivity, cost efficiency, and compatibility with automated monitoring, ISEs are increasingly being employed in environmental and industrial applications. Yet, their polymeric membranes make them particularly prone to hydrophobic fouling, resulting in signal instability and shortened lifetime.

Fouling on ISEs leads to deterioration in selectivity, stability, and sensitivity, as well as reduced reproducibility and detection limits. The underlying mechanisms involve nonspecific adsorption and electrode passivation through hydrophilic, hydrophobic, or electrostatic interactions—either reversible or irreversible. Proteins, for instance, can cause potential drifts, while biofilms may reduce sensitivity or distort local conditions, such as pH.

Various techniques are available to detect and quantify fouling on ISEs. Electrochemical methods—such as potentiometry, amperometry, and electrochemical impedance spectroscopy (EIS)—provide indirect insights into surface changes, whereas physical and microscopic techniques, including quartz crystal microbalance (QCM), scanning electron microscopy (SEM), and X-ray diffraction (XRD), enable direct surface characterization. Complementary approaches, such as membrane cleaning followed by analysis of removed foulants, yield additional information on fouling composition and intensity. In aquaculture systems, in situ implementation of electrochemical monitoring allows predictive maintenance scheduling and enhances the long-term stability of automated sensor networks.

To counteract electrode drift and surface deterioration, ISEs can be preconditioned in a sample matrix or rinsed in a low-analyte solution. For heavily fouled electrodes, crystalline ISEs (e.g., glass pH sensors) often require polishing, while polymeric ISEs typically demand membrane replacement. Cleaning strategies include mechanical (e.g., wipers or brushes), chemical (e.g., acids, EDTA, or UV treatment), and physical (e.g., ultrasound) methods. Regular cleaning and recalibration remain essential for maintaining long-term measurement accuracy. In aquaculture applications, cleaning routines must achieve effective fouling removal while preventing toxic residues that could endanger cultured organisms.

Preventive antifouling strategies further reduce fouling formation by optimizing sensor materials, applying surface modifications, and integrating process control measures such as sample pretreatment or intermittent cleaning cycles. Coatings and thin films are among the most common antifouling methods, while biomimetic concepts, such as limiting light exposure to inhibit algal growth, offer passive yet effective protection. Material design focusing on hydrophilic, hydrophobic, or amphiphilic surface properties has proven particularly useful in minimizing foulant adhesion. Future developments should emphasize sustainable antifouling concepts that support continuous monitoring in aquaculture while ensuring environmental safety and animal welfare.

Antifouling coatings—including hydrophobic, hydrophilic, amphiphilic, and biocidal systems—are well established in marine applications. However, traditional coatings such as those containing tributyltin (TBT) have raised ecological and regulatory concerns due to their toxicity and persistence. This has spurred research into environmentally benign alternatives, including natural, zwitterionic, and self-cleaning coatings. Surface modifications enhancing hydrophilicity, zwitterionic character, or superhydrophobicity have shown significant potential in improving ISE longevity and performance. Increasingly, hybrid and bioinspired antifouling systems that combine physical structuring with chemical functionality are being developed to ensure sustainable, long-term sensor operation. Such approaches are especially relevant for aquaculture, where maintaining precise and reliable monitoring under high biological activity must align with strict ecological and regulatory requirements.

## 11. Conclusions

ISEs are indispensable tools for real-time water quality monitoring in environmental, industrial, and aquaculture applications. Their high sensitivity, selectivity, and cost effectiveness make them particularly suitable for continuous process control. However, their long-term performance is significantly constrained by fouling and biofouling, which impair sensitivity, stability, and measurement accuracy, ultimately shortening sensor lifespan.

This challenge is especially pronounced in aquaculture systems, where sensors operate continuously in biologically active and nutrient-rich environments. Under such conditions, fouling not only causes signal drift and response delays but can also compromise the regulation of critical parameters, such as pH, dissolved oxygen, and ammonium, that directly influence animal health and production efficiency.

This review has summarized the main mechanisms of fouling, established detection and regeneration methods, and emerging antifouling strategies for ISEs. Regenerative techniques—including mechanical, chemical, acoustic, and electrochemical cleaning—have proven effective in restoring sensor performance, although periodic recalibration and maintenance remain essential for sustained reliability. Preventive approaches, in contrast, offer a more sustainable path by reducing fouling formation at its source through the use of optimized materials, surface modifications, and advanced coatings.

Recent advances in carbon-based sensor materials, bioinspired surface architectures, and environmentally benign antifoulants highlight a growing trend toward sustainable and durable sensor technologies. These developments are particularly relevant for aquaculture monitoring, where minimizing maintenance requirements and preventing chemical contamination are essential for economic efficiency and ecological safety.

Future research should focus on hybrid systems that integrate regeneration and antifouling functionalities within the same sensor platform, enabling self-cleaning, adaptive, and autonomous ISEs suitable for long-term deployment in challenging aquatic environments. Incorporating such smart sensors into RAS would facilitate predictive water quality management, enhance resource efficiency, and strengthen system resilience.

Ultimately, combining physical, chemical, and material-based innovations will remain key to achieving reliable, accurate, and sustainable sensor performance. The integration of smart antifouling materials with in situ regeneration capabilities represents a decisive step toward the next generation of intelligent environmental and aquaculture sensors.

## 12. Materials and Methods

A limited use of large-language-model (LLM)-based assistance (e.g., ChatGPT) was restricted solely to linguistic editing tasks, including stylistic polishing, grammar improvement, and minor text shortening. All scientific content, interpretations, analyses, and conclusions were conceived, generated, and validated exclusively by the authors. The authors carefully verified and corrected all AI-assisted edits to ensure factual accuracy and adherence to scientific standards.

## Figures and Tables

**Table 1:** Comparison of different methods to detect water quality parameters and their advantages and disadvantages regarding performance.

Method	Advantage	Disadvantage
chromatography, mass spectrometry	high accuracy, multi-component analysis	offline, expensive, time-shifted, geographically shifted
immunoassays, PCR, culture-based techniques	high selectivity, pathogens	slow, expensive, sensitive, non-continuous
biosensors, nano sensors	high sensitivity, molecular detection, (inline)	instability, fouling, complex, short lifespan

“High”, “medium”, and “low” reflect relative performance reported across the literature, based on comparative sensitivity, reproducibility, maintenance frequency, and operational robustness of the respective methods. They represent aggregated evidence from multiple studies instead of absolute numerical thresholds.

**Table 2:** Advantages and disadvantages (regarding performance) of different methods to detect water quality parameters.

Sensor	Advantage	Disadvantage
mechanical transduction	high sensitivity for mass, pressure, and surface changes	drifts through vibration, temperature, fouling, difficult to miniaturize
optical transduction	very high sensitivity, no electromagnetic interferences	fouling of optics, high energy consumption, expensive optics
electrochemical transduction	ideal for water quality parameters, easy to miniaturize, low energy consumption, cheap	fouling, ageing of electrodes, drifts (reference electrode)
electrical conduction	easy, cheap, easy to miniaturize	low selectivity, highly temperature- or salinity-dependent, fouling

“High” and “low” reflect relative performance reported across the literature, based on comparative sensitivity, reproducibility, maintenance frequency, and operational robustness of the respective methods. They represent aggregated evidence from multiple studies instead of absolute numerical thresholds.

**Table 3:** Summary of reported fouling effects on ISEs.

Fouling Effects	Proteins	Thrombus	Microorganisms	Biofilms	Lipids	Oils	Organic Matter
potential drift	x		x		x		x
decrease in sensitivity		x		x		x	x
decrease in stability		x		x		x	
shortened lifetime		x				x	
increased measurement error		x					
increased response time				x			
deterioration of detection limit		x					
deterioration of selectivity					x		
microbial adhesion	x						
biofilm formation			x				

Note: This synthesized table is based on qualitative descriptions from the literature (Qi et al., 2022), no tables or figures from the original sources were reproduced or adapted [4].

**Table 4:** Comparison of methods to detect fouling on ISEs regarding sensitivity, reproducibility, early detection, inline, and suitability for aquacultures and notes.

Method	Sensitivity	Reproducibility	Early Detection	Inline	Suitable for Aquacultures	Notes
QCM	very high	high	Yes	limited	low	detects mass increase, delicate
SPR	very high	very high	yes	limited	low	expensive, highly sensitive
potentiometry	medium	medium	limited	excellent	very high	detects drift in situ
EIS	high	high	excellent	partial	high	detects impedance changes from biofilm
chemical dying	medium-high	medium	indirect	no	medium	stains biofilms, lab method
UV/VIS	low-medium	medium	indirect	no	low	optical turbidity flawed in aquaculture
fluorescence microscopy	high	medium	yes	no	medium	confocal, viable for lab validation
SEM	very high	very high	no	no	low	destructive, ex situ
AFM	very high	high	no	no	low	topography of membrane surface
Raman	high	high	yes	no	low	fouling chemical identification
XPS/XRD	high	high	no	no	low	detailed chemical/fouling composition
chronopotentiometry	high	medium	yes	limited	high	detects resistance increase
CV	medium	medium	limited	no	medium	reference-electrode fouling
amperometry	medium	medium	yes	possible	high	DO-sensor fouling assessment
conductometry	medium	medium	yes	yes	high	early biofilm proxy via conductivity

“High”, “medium”, and “low” reflect relative performance reported across the literature, based on comparative sensitivity, reproducibility, maintenance frequency, and operational robustness of the respective methods. They represent aggregated evidence from multiple studies instead of absolute numerical thresholds.

**Table 5:** Comparison of methods to detect fouling on ISE regarding response time, maintenance frequency, cost, tolerance to salinity and temperature, and durability.

Method	Response Time	Maintenance Frequency	Cost	Tolerance to Salinity	Tolerance to Temperature	Durability
QCM	fast	high	high	low	low	low
SPR	fast	high	very high	low	low	low
potentiometry	very fast	low	low	high	high	high
EIS	fast	low	medium	high	high	high
chemical dying	slow	medium	low	high	medium	medium
UV/VIS	fast	low	low	medium	medium	medium
fluorescence microscopy	medium	medium	high	low	low	medium
SEM	slow	high	high	indifferent	indifferent	low
AFM	slow	high	high	indifferent	indifferent	low
Raman	medium	medium	high	medium	medium	medium
XPS/XRD	slow	high	high	indifferent	indifferent	low
chronopotentiometry	fast	low	low	high	high	high
CV	fast	low	low	high	high	high
amperometry	fast	medium	low-medium	high	medium	high
conductometry	very fast	low	low	high	high	high

“High”, “medium”, and “low” reflect relative performance reported across the literature, based on comparative sensitivity, reproducibility, maintenance frequency, and operational robustness of the respective methods. They represent aggregated evidence from multiple studies instead of absolute numerical thresholds.

**Table 6:** Comparison of methods to clean fouling on ISEs.

Method	Target Fouling	Effectiveness	Maintenance Frequency	Equipment Required	Risk to Membrane	Suitability in RAS	Notes
preconditioning	reversible	medium	high	none	low	very high	stabilizes baseline
routine rinsing	reversible	low-medium	high	none	low	very high	removes fresh biofilm
mechanical wiping	reversible/early irreversible	high	medium	wiper/brush	medium	high	widely used in RAS
polishing	irreversible	high	low	polishing pads	high	low	only for glass/solid electrodes
water jet	reversible	medium	medium	pump/nozzle	low	medium	good for debris removal
EDTA	inorganic fouling	high	medium	chemical	medium	high	removes metallic deposits
ascorbic acid	sulphate/oxide	medium	medium	chemical	low	medium	mild cleaning agent
perchloric acid	heavy inorganic	very high	low	chemical	very high	low	aggressive, risk of membrane damage
mild organic acids	biofilm	medium	high	chemical	low	high	safe for aquaculture
ultrasound	biofilm	medium-high	medium	ultrasonic transducer	low	high	promising for RAS
acoustic vibration	larvae/soft fouling	medium	medium	actuator	low	medium	prevents settlement
electrochemical cleaning	biofilm/organic	high	low-medium	electronics	medium	high	ideal for SC-ISEs
integrated hybrid	all	very high	low	multiple	medium	very high	best long-term strategy

“High”, “medium”, and “low” reflect relative performance reported across the literature, based on comparative sensitivity, reproducibility, maintenance frequency, and operational robustness of the respective methods. They represent aggregated evidence from multiple studies instead of absolute numerical thresholds.

**Table 7:** Comparison of cleaning agents and their compatibility to different materials used in ISE.

Cleaning Agent	PVC Membrane	Polyurethane	PTFE-Doped Membrane	Glass	Enamel	Diamond	Carbon	Ag/AgCl Reference
EDTA	medium	medium	high	safe	safe	safe	safe	unsafe (chelates Ag^+^)
ascorbic acid	safe	safe	safe	safe	safe	safe	safe	safe
perchloride acid	not safe	not safe	not safe	limited	safe	safe	safe	not safe
mild organic acids	safe	safe	safe	safe	safe	safe	safe	safe
sodium carbonate	safe	safe	safe	safe	safe	safe	safe	safe
mechanical polishing	not safe	not safe	not safe	safe	safe	safe	medium	not safe
water jet	safe	safe	safe	safe	safe	safe	safe	safe
ultrasound	safe	safe	safe	safe	safe	safe	safe	Safe
electrochemical pulsing	medium	medium	medium	safe	safe	safe	safe	medium

“High”, “medium”, and “safe/not safe” reflect relative performance reported across the literature, based on comparative sensitivity, reproducibility, maintenance frequency, and operational robustness of the respective methods. They represent aggregated evidence from multiple studies instead of absolute numerical thresholds.

**Table 8:** Comparison between possible materials for ISE construction.

Material	Lifetime	Sensor Performance	Fouling Resistance	Notes
glass	short-term	high drift	moderate	Standard pH sensor
enamelled steel	can exceed 100 years	low impact on response time	high	Excellent for harsh media
carbon-composite/nanoporous carbon	long-term	moderate drift	superior	-
diamond	extreme long-term	fast response time	very high	Excellent for harsh media
antimony	/	/	enhanced	Robust
metals	long-term	/	high	-

E.g., “high”, “enhanced”, and “superior” reflect relative performance reported across the literature, based on comparative sensitivity, reproducibility, maintenance frequency, and operational robustness of the respective methods. They represent aggregated evidence from multiple studies instead of absolute numerical thresholds.

**Table 9:** Overview of antifoulant types, sensor applications, and related findings reported in the literature.

Type	Sensor Body	Sensor Tip	Antifoulant	Note	Reference
natural alternatives	-	-	marine-derived compounds (e.g., algae extract)	environmentally benign; biodegradable	[69]
foul-release materials	maritime sensors	-	silicones, silicone hydrogels, fluoropolymers	non-toxic; prevent adhesion	[69]
special hydrogels/ bacteria	-	-	hydrogels, bacteria and their extracellular components	complex maintenance; variable performance	[71]
polymeric membrane coatings	ISEs	membrane	silicones, silicone hydrogels, fluoropolymers	synthetic coatings improving fouling resistance	[4]
photocatalytic coatings	ISEs	surface	TiO_2_ (anatase)	UV-activated degradation of organic matter; superhydrophilic surface	[76,92]
toxic metal comparison	-	-	-	copper toxicity is pH-dependent; unsuitable for pH sensors	[118]
copper/zinc-based coatings	biofouling prevention		Cu–Zn-based coatings	average biofouling reduction ~59%	[84]
booster biocides	marine paints		secondary biocides (organotin-free)	enhance copper-based antifouling effectiveness; compensate for algal tolerance	[118]
self-cleaning zwitterionic coatings	ISEs	surface	zwitterionic polymer	prevents oil fouling; effective on calcium ISEs	[89]

**Table 10:** Comparison of conventional additives vs. ionic liquids.

Antifouling Method	Response Time	Maintenance Frequency	Drift Impact	Applicability (Salinity and Temperature)	Durability
conventional additives (plasticizers, lipophilic salts)	fast	frequent calibration and cleaning	high (leaching and ageing)	moderate (membrane destabilization)	moderate
ionic liquid (1-hexyl-3-methylimidazolium	unchanged or slightly improved	reduced (less leaching)	low → improved slope stability	good (improved stability)	high
trihexyltetradecylphosphonium	fast	very low (stable over months)	extremely low (−58.9 vs. −60.1 mV/decade after 4 months)	very good (high thermal and chemical stability)	very high

“High”, “medium”, and “low” reflect relative performance reported across the literature, based on comparative sensitivity, reproducibility, maintenance frequency, and operational robustness of the respective methods. They represent aggregated evidence from multiple studies instead of absolute numerical thresholds.

**Table 11:** Comparison of antifouling coating types regarding response time impact on sensors, durability, drift impact, RAS suitability, and notes.

Coating Type	Response Time Impact	Durability	Drift Impact	RAS Suitability	Notes
silicone foul-release	slightly slower (5–20%)	high	low	very good	used in marine industry
fluoropolymers	negligible	high	very low	excellent	non-stick and chemical inert
zwitterionic polymer coatings	none-low	medium-high	very low	good	best against protein fouling
hydrogel coatings	moderate	low-medium	medium	low	swelling problematic in RAS
TiO_2_ photocatalytic	none	high (needs UV)	very low	medium	requires UV activation
biomimetic microtextures	none	medium	low	limited	hard to manufacture
biocide coatings	none	variable	very low	poor (toxicity risk)	not recommended for aquaculture

“High”, “medium”, and “low” reflect relative performance reported across the literature, based on comparative sensitivity, reproducibility, maintenance frequency, and operational robustness of the respective methods. They represent aggregated evidence from multiple studies instead of absolute numerical thresholds.

## Data Availability

Not applicable.

## Abbreviations

The following abbreviations are used in this manuscript:

AFM	Atomic Force Microscopy
Ag/AgCl	Silver/Silver Chloride
BMA	Butyl Methacrylate
CV	Cyclic Voltammetry
DC	Direct Current
DO	Dissolved Oxygen
EC	Electrical Conductivity
EDTA	Ethylenediaminetetraacetic Acid
EHMA	2-Ethylhexyl Methacrylate
EIS	Electrochemical Impedance Spectroscopy
ESCA	Electron Spectroscopy for Chemical Analysis
FESEM	Field Emission Scanning Electron Microscopy
HClO_4_	Perchloric Acid
IO_3_^−^	Iodate Ion
ISE	Ion-Selective Electrode
ISM	Ion-Selective Membrane
KCl	Potassium Chloride
LOD	Limit Of Detection
MPC	2-Methacryloyloxyethyl Phosphorylcholine
NaCl	Sodium Chloride
NH_4_^+^	Ammonium Ion
NO_3_^−^	Nitrate Ion
NTU	Nephelometric Turbidity Units
ORP	Oxidation–Reduction Potential
PCR	Polymerase Chain Reaction
PEG	Polyethylene Glycol
PEEK	Polyether Ether Ketone
pH	Pondus Hydrogenii, Potential Hydrogenii
PTFE	Polytetrafluoroethylene
PVC	Polyvinyl Chloride
QCM	Quartz Crystal Microbalance
RAS	Recirculating Aquaculture System
ROS	Reactive Oxygen Species
SC-ISE	Solid-Contact Ion-Selective Electrode
TiO_2_	Titanium Oxide
UV/VIS	Ultraviolet/Visible Spectroscopy

## References

[1] Verma D.K., Satyaveer N.K.M., Kumar P., Jayaswa R. (2022). Important water quality parameters in aquaculture: An overview. Agric. Environ..

[2] Lindholm-Lehto P. (2023). Water quality monitoring in recirculating aquaculture systems. Aquac. Fish Fish..

[3] Parisi C., Sandonnini J., Coppola M.R., Madonna A., Abdel-Gawad F.K., Sivieri E.M., Guerriero G. (2022). Biocide vs. Eco-Friendly Antifoulants: Role of the Antioxidative Defence and Settlement in Mytilus galloprovincialis. JMSE.

[4] Qi L., Liang R., Jiang T., Qin W. (2022). Anti-fouling polymeric membrane ion-selective electrodes. TrAC Trends Anal. Chem..

[5] Liu Z., Zhao H., Jiang T., Qin W. (2025). Influences of marine biofouling on the potential responses of polymeric membrane ion-selective electrodes. Sens. Actuators B Chem..

[6] Edokpayi J.N., Odiyo J.O., Durowoju O.S., Tutu H. (2017). Impact of Wastewater on Surface Water Quality in Developing Countries: A Case Study of South Africa. Water Quality.

[7] Madhura L., Singh S., Kanchi S., Sabela M., Bisetty K., Inamuddin (2019). Nanotechnology-based water quality management for wastewater treatment. Environ. Chem. Lett..

[8] Asghar M.Z. (2018). Comparative assessment of physico-chemical parameters of waste water effluents from different industries in Lahore, Pakistan. Proc. Int. Acad. Ecol. Environ. Sci..

[9] Hutton P.H., Roy S.B., Krasner S.W., Palencia L. (2022). The Municipal Water Quality Investigations Program: A Retrospective Overview of the Program’s First Three Decades. Water.

[10] Kumar A.A., Jaison J., Prabakaran K., Nagarajan R., Chan Y.S. (2016). Water quality monitoring: A comparative case study of municipal and Curtin Sarawak’s lake samples. IOP Conf. Ser. Mater. Sci. Eng..

[11] Agostinho A.A., Alves D.C., Gomes L.C., Dias R.M., Petrere M., Pelicice F.M. (2021). Fish die-off in river and reservoir: A review on anoxia and gas supersaturation. Neotrop. Ichthyol..

[12] Boguniewicz-Zablocka J., Klosok-Bazan I., Naddeo V. (2019). Water quality and resource management in the dairy industry. Environ. Sci. Pollut. Res. Int..

[13] Tüfekçi N., Sivri N., Toroz İ. (2007). Pollutants of Textile Industry Wastewater and Assessment of its Discharge Limits by Water Quality Standards. Turk. J. Fish. Aquat. Sci..

[14] Humpert D., Ebrahimi M., Czermak P. (2016). Membrane Technology for the Recovery of Lignin: A Review. Membranes.

[15] Su X., Sutarlie L., Loh X.J. (2020). Sensors, Biosensors, and Analytical Technologies for Aquaculture Water Quality. Research.

[16] Antonucci F., Costa C. (2020). Precision aquaculture: A short review on engineering innovations. Aquac. Int..

[17] Lafont M., Dupont S., Cousin P., Vallauri A., Dupont C. (2019). Back to the future: IoT to improve aquaculture: Real-time monitoring and algorithmic prediction of water parameters for aquaculture needs. Proceedings of the 2019 Global IoT Summit (GIoTS).

[18] Al-Kaabi A., Al-Sulaiti H., Al-Ansari T., Mackey H.R. (2021). Assessment of water quality variations on pretreatment and environmental impacts of SWRO desalination. Desalination.

[19] Boyd C.E., Jeney G. (2017). Chapter 6—General Relationship Between Water Quality and Aquaculture Performance in Ponds. Fish Diseases: Prevention and Control Strategies.

[20] Elsherif S.M., Wang S., Taha A.F., Sela L., Giacomoni M.H., Abokifa A.A. (2023). Control-theoretic modeling of multi-species water quality dynamics in drinking water networks: Survey, methods, and test cases. Annu. Rev. Control.

[21] Hasby A.R., Fadhlurrahman F., Nugrahardo H.A., Assiddiqi T.D., Astuti A.D., Kurniawan A. (2024). Assessing Permeate Water Quality in Recirculating Aquaculture Systems Using Nanofiltration Membrane Technology and Various Pre-Treatment Configurations. BIO Web Conf..

[22] Whetstone J.M., Treece G.D., Browdy C.L., Stokes A.D. (2000). Opportunities and Constraints in Marine Shrimp Farming.

[23] Cho J.H., Seok Sung K., Ryong Ha S. (2004). A river water quality management model for optimising regional wastewater treatment using a genetic algorithm. J. Environ. Manag..

[24] Karthick G.S., Sridhar M., Pankajavalli P.B. (2020). Internet of Things in Animal Healthcare (IoTAH): Review of Recent Advancements in Architecture, Sensing Technologies and Real-Time Monitoring. SN Comput. Sci..

[25] Kalid N., Zaidan A.A., Zaidan B.B., Salman O.H., Hashim M., Muzammil H. (2017). Based Real Time Remote Health Monitoring Systems: A Review on Patients Prioritization and Related “Big Data” Using Body Sensors information and Communication Technology. J. Med. Syst..

[26] Blaen P.J., Khamis K., Lloyd C.E.M., Bradley C., Hannah D., Krause S. (2016). Real-time monitoring of nutrients and dissolved organic matter in rivers: Capturing event dynamics, technological opportunities and future directions. Sci. Total Environ..

[27] Abu-Absi N.R., Kenty B.M., Cuellar M.E., Borys M.C., Sakhamuri S., Strachan D.J., Sakhamuri S., Strachan D.J., Hausladen M.C., Li Z.J. (2011). Real time monitoring of multiple parameters in mammalian cell culture bioreactors using an in-line Raman spectroscopy probe. Biotechnol. Bioeng..

[28] Bourgeois W., Burgess J.E., Stuetz R.M. (2001). On-line monitoring of wastewater quality: A review. J. Chem. Technol. Biotechnol..

[29] Vijayakumar N., Ramya R. (2015). The real time monitoring of water quality in IoT environment. Proceedings of the International Conference on Innovations in Information, Embedded and Communication Systems (ICIIECS).

[30] Parra L., Lloret G., Lloret J., Rodilla M. (2018). Physical Sensors for Precision Aquaculture: A Review. IEEE Sens. J..

[31] Delgado A., Briciu-Burghina C., Regan F. (2021). Antifouling Strategies for Sensors Used in Water Monitoring: Review and Future Perspectives. Sensors.

[32] Kruse P. (2018). Review on water quality sensors. J. Phys. D Appl. Phys..

[33] Dimeski G., Badrick T., John A.S. (2010). Ion Selective Electrodes (ISEs) and interferences—A review. Clin. Chim. Acta Int. J. Clin. Chem..

[34] Cho W.J., Kim D.-W., Jung D.H., Cho S.S., Kim H.-J. (2016). An Automated Water Nitrate Monitoring System Based on Ion-Selective Electrodes. J. Biosyst. Eng..

[35] Ahmadzadeh S., Rezayi M., Faghih-Mirzaei E., Yoosefian M., Kassim A. (2015). Highly Selective Detection of Titanium (III) in Industrial Waste Water Samples Using Meso-octamethylcalix[4]pyrrole-Doped PVC Membrane Ion-Selective Electrode. Electrochim. Acta.

[36] Guziński M., Lisak G., Kupis J., Jasiński A., Bocheńska M. (2013). Lead(II)-selective ionophores for ion-selective electrodes: A review. Anal. Chim. Acta.

[37] Wardak C., Pietrzak K., Morawska K., Grabarczyk M. (2023). Ion-Selective Electrodes with Solid Contact Based on Composite Materials: A Review. Sensors.

[38] Kim H.-J., Kim W.-K., Roh M.-Y., Kang C.-I., Park J.-M., Sudduth K.A. (2013). Automated sensing of hydroponic macronutrients using a computer-controlled system with an array of ion-selective electrodes. Comput. Electron. Agric..

[39] Jung D.H., Kim H.-J., Choi G.L., Ahn T.-I., Son J.-E., Sudduth K.A. (2015). Automated lettuce nutrient solution management using an array of ion-selective electrodes. Trans. ASABE.

[40] Summers J.K. (2020). Water Quality: Science, Assessments and Policy.

[41] Troudt B.K., Rousseau C.R., Dong X.I.N., Anderson E.L., Bühlmann P. (2022). Recent progress in the development of improved reference electrodes for electrochemistry. Anal. Sci..

[42] Westbroek P., Kiekens P., Westbroek P., Priniotakis G., Kiekens P. (2005). 3—Probes for pH measurement and simultaneous cellulose removal and bleaching of textiles with enzymes. Analytical Electrochemistry in Textiles.

[43] Golan R., Gavrieli I., Lazar B., Ganor J. (2014). The determination of pH in hypersaline lakes with a conventional combination glass electrode. Limnol. Oceanogr. Methods.

[44] Nisah K., Rahmi R., Ramli M., Idroes R., Alva S., Iqhrammullah M., Safitri E. (2022). Optimization of Castor Oil-Based Ion Selective Electrode (ISE) with Active Agent 1,10-Phenanthroline for Aqueous Pb^2+^ Analysis. Membranes.

[45] Kitazumi Y. (2022). Recent development of ion-selective electrodes. Anal. Sci..

[46] Vogel S., Emmerich K., Schröter I., Bönecke E., Schwanghart W., Rühlmann J., Kramer E., Gebbers R. (2023). The effect of soil moisture content and soil texture on fast in situ pH measurements with two types of robust ion-selective electrodes. EGUsphere.

[47] Kumar V., Suri R., Mittal S. (2023). Review on new ionophore species for membrane ion selective electrodes. J. Iran. Chem. Soc..

[48] Mohan C., Robinson J., Negi A. (2023). Ion-Selective Electrode (ISE) Based on Polyvinyl Chloride Membrane Formed from Heterocyclic Quinazoline Compounds as Ionophore material. Eng. Proc..

[49] Li Y., Li J., Qin W. (2022). All-Solid-State Polymeric Membrane Ion-Selective Electrodes Based on NiCo_2_S_4_ as a Solid Contact. Anal. Chem..

[50] Criscuolo F., Hanitra M.I.N., Taurino I., Carrara S., de Micheli G. (2021). All-Solid-State Ion-Selective Electrodes: A Tutorial for Correct Practice. IEEE Sens. J..

[51] Yrjänä V., Mousavi Z., Sokalski T., Joon N., Leito I., Bobacka J. (2025). Influence of electrode body material on the analytical behaviour of solid-contact ion-selective electrodes. Talanta.

[52] Zhou L., Boyd C.E. (2016). Comparison of Nessler, phenate, salicylate and ion selective electrode procedures for determination of total ammonia nitrogen in aquaculture. Aquaculture.

[53] Eze E., Halse S., Ajmal T. (2021). Developing a Novel Water Quality Prediction Model for a South African Aquaculture Farm. Water.

[54] Ozer T., Agir I., Borch T. (2024). Water monitoring with an automated smart sensor supported with solar power for real-time and long range detection of ferrous iron. Analyst.

[55] Bratovčić A., Odobašić A., Ćatić S. (2009). The Advantages of the Use of Ion—Selective Potentiometry in Relation to UV/VIS Spectroscopy. Agric. Conspec. Sci..

[56] Kaelin D., Rieger L., Eugster J., Rottermann K., Bänninger C., Siegrist H. (2008). Potential of in-situ sensors with ion-selective electrodes for aeration control at wastewater treatment plants. Water Sci. Technol. A J. Int. Assoc. Water Pollut. Res..

[57] Soda Y., Bakker E. (2021). Ionic strength-independent potentiometric cation concentration sensing on paper using a tetrabutylammonium-based reference electrode. Sens. Actuators B Chem..

[58] Qi L., Jiang T., Liang R., Qin W. (2021). Polymeric membrane ion-selective electrodes with anti-biofouling properties by surface modification of silver nanoparticles. Sens. Actuators B Chem..

[59] Guo Y., Wang C., Han G., Nyein H.Y.Y. (2024). Wearable ion-selective sensors with rapid conditioning and extended stability achieved through modulation of water and ion transport. Biosens. Bioelectron. X.

[60] Janiszewska J., Balcerzak M. (2013). Analytical Problems with the Evaluation of Human Exposure to Fluorides from Tea Products. Food Anal. Methods.

[61] Alva S., Septyanda P., Burhanudin A., Khaerudini D.S., Jenie S.N.A., Sundari R., Suhud K. (2023). Development of Nitrate-Ion Selective Electrode (NO_3_-ISE) Based on Carbon from Disposal Battery. ECS J. Solid State Sci. Technol..

[62] He Z., Lin E., Wen Y., Hu S., Chen H., Zhang P., Yang Y., Guo W. (2025). Ammonia Nitrogen Detection in Acidic Solutions: An Ammonium ISE Coupled With pH Precompensation. IEEE Trans. Instrum. Meas..

[63] Morawska K., Wardak C. (2024). Application of ionic liquids in ion-selective electrodes and reference electrodes: A review. Chemphyschem.

[64] Nussbaum R., Jeanneret S., Bakker E. (2024). Increasing the Sensitivity of pH Glass Electrodes with Constant Potential Coulometry at Zero Current. Anal. Chem..

[65] Cecconi F., Reifsnyder S., Sobhani R., Cisquella-Serra A., Madou M., Rosso D. (2020). Functional behaviour and microscopic analysis of ammonium sensors subject to fouling in activated sludge processes. Environ. Sci. Water Res. Technol..

[66] Grzegorczyk M., Pogorzelski S.J., Pospiech A., Boniewicz-Szmyt K. (2018). Monitoring of Marine Biofilm Formation Dynamics at Submerged Solid Surfaces With Multitechnique Sensors. Front. Mar. Sci..

[67] Lyu Y., Gan S., Bao Y., Zhong L., Xu J., Wang W., Liu Z., Ma Y., Yang G., Niu L. (2020). Solid-Contact Ion-Selective Electrodes: Response Mechanisms, Transducer Materials and Wearable Sensors. Membranes.

[68] Flemming H.-C. (2002). Biofouling in water systems-cases, causes and countermeasures. Appl. Microbiol. Biotechnol..

[69] Dafforn K.A., Lewis J.A., Johnston E.L. (2011). Antifouling strategies: History and regulation, ecological impacts and mitigation. Mar. Pollut. Bull..

[70] Zhou L., Li X., Zhu B., Su B. (2022). An Overview of Antifouling Strategies for Electrochemical Analysis. Electroanalysis.

[71] Whelan A., Regan F. (2006). Antifouling strategies for marine and riverine sensors. J. Environ. Monit. JEM.

[72] Fusetani N. (2011). Antifouling marine natural products. Nat. Prod. Rep..

[73] Compère C., Bellon-Fontaine M.-N., Bertrand P., Costa D., Marcus P., Poleunis C., Pradier C.M., Rondot B., Walls M.G. (2001). Kinetics of conditioning layer formation on stainless steel immersed in seawater. Biofouling.

[74] Cattò C., Corte L., Roscini L., Cardinali G., Villa F., Cappitelli F. (2022). Metabolomic and Proteomic Changes in Candida albicans Biofilm in Response to Zosteric Acid Treatment. Int. J. Mol. Sci..

[75] Gu Y., Yu L., Mou J., Wu D., Xu M., Zhou P., Ren Y. (2020). Research Strategies to Develop Environmentally Friendly Marine Antifouling Coatings. Mar. Drugs.

[76] Gizer G., Önal U., Ram M., Sahiner N. (2022). Biofouling and Mitigation Methods: A Review. Biointerface Res. Appl. Chem..

[77] Fitridge I., Dempster T., Guenther J., de Nys R. (2012). The impact and control of biofouling in marine aquaculture: A review. Biofouling.

[78] Tolker-Nielsen T., Ghannoum M., Parsek M., Whiteley M., Mukherjee P.K. (2015). Biofilm Development. Microbial Biofilms.

[79] Munro W.A., Thomas C., Simpson I., Shaw J., Dodgson J. (1996). Deterioration of pH electrode response due to biofilm formation on the glass membrane. Sens. Actuators B Chem..

[80] Delauney L., Compère C., Lehaitre M. (2010). Biofouling protection for marine environmental sensors. Ocean Sci..

[81] Bdiri M., Larchet C., Dammak L. (2020). A Review on Ion-exchange Membranes Fouling and Antifouling During Electrodialysis Used in Food Industry: Cleanings and Strategies of Prevention. Chem. Afr..

[82] Li Y., Han R., Chen M., Zhang L., Wang G., Luo X. (2021). Bovine Serum Albumin-Cross-Linked Polyaniline Nanowires for Ultralow Fouling and Highly Sensitive Electrochemical Protein Quantification in Human Serum Samples. Anal. Chem..

[83] Kerr A., Cowling M.J., Beveridge C.M., Smith M.J., Parr A., Head R.M., Davenport J., Hodgkiess T. (1998). The early stages of marine biofouling and its effect on two types of optical sensors. Environ. Int..

[84] Venkatesan R., Kadiyam J., SenthilKumar P., Lavanya R., Vedaprakash L. (2017). Marine Biofouling on Moored Buoys and Sensors in the Northern Indian Ocean. Mar. Technol. Soc. J..

[85] Hanssen B.L., Siraj S., Wong D.K. (2016). Recent strategies to minimise fouling in electrochemical detection systems. Rev. Anal. Chem..

[86] Lisak G., Arnebrant T., Lewenstam A., Bobacka J., Ruzgas T. (2016). In Situ Potentiometry and Ellipsometry: A Promising Tool to Study Biofouling of Potentiometric Sensors. Anal. Chem..

[87] Alsakran A.A., Elmessery W.M., Szűcs P., Eid M.H., Shams M.Y., Hassan E., El-Hafeez T.A., Mahmoud S.F., Saleh D.I., AlQthanin R.N. (2025). Enhancing interpretability and explainability for fish farmers: Decision tree approximation of DDPG for RAS control. Aquacult. Int..

[88] Canali C., Larsen L.B., Martinsen Ø.G., Heiskanen A. (2015). Conductometric analysis in bio-applications: A universal impedance spectroscopy-based approach using modified electrodes. Sens. Actuators B Chem..

[89] Qi L., Jiang T., Liang R., Qin W. (2021). Enhancing the Oil-Fouling Resistance of Polymeric Membrane Ion-Selective Electrodes by Surface Modification of a Zwitterionic Polymer-Based Oleophobic Self-Cleaning Coating. Anal. Chem..

[90] Zhao P., Patamia E.D., Andrew T.L. (2023). Strategies to combat the fouling and surface texture issues associated with fabric-based colorimetric sensors. Sens. Actuators B Chem..

[91] Radu A., Anastasova-Ivanova S., Paczosa-Bator B., Danielewski M., Bobacka J., Lewenstam A., Diamond D. (2010). Diagnostic of functionality of polymer membrane-based ion selective electrodes by impedance spectroscopy. Anal. Methods.

[92] Liu T., Liang R., Qin W. (2023). Anti-fouling TiO_2_-Coated Polymeric Membrane Ion-Selective Electrodes with Photocatalytic Self-Cleaning Properties. Anal. Chem..

[93] Zhu F., Xue Y., Ji W., Li X., Ma W., Yu P., Jiang Y., Mao L. (2023). Galvanic Redox Potentiometry for Fouling-Free and Stable Serotonin Sensing in a Living Animal Brain. Angew. Chem. Int. Ed..

[94] Lee J.-U., Park S.Y., Lee K., Farzana S., Chun H.H., Shin B.-S., Lee P.C. (2024). Laser micro/nano structuring of three-dimensional porous gradient graphene: Advanced heater for antibacterial surfaces and ion-selective electrode for sweat sensing. Carbon.

[95] Esmeryan K.D., Castano C.E., Abolghasemibizaki M., Mohammadi R. (2017). An artful method for in-situ assessment of the anti-biofouling potential of various functional coatings using a quartz crystal microbalance. Sens. Actuators B Chem..

[96] Wang J., Jiang M., Lu F. (1998). Electrochemical quartz crystal microbalance investigation of surface fouling due to phenol oxidation. J. Electroanal. Chem..

[97] Men H., Mu S., Peng Y., An L. (2009). Detection of Microbes in Natural Industrial Cooling Water by Conductometric Method. Proceedings of the International Conference on Measuring Technology and Mechatronics Automation, ICMTMA.

[98] Kondratyeva Y.O., Tolstopjatova E.G., Kirsanov D.O., Mikhelson K.N. (2020). Chronoamperometric and coulometric analysis with ionophore-based ion-selective electrodes: A modified theory and the potassium ion assay in serum samples. Sens. Actuators B Chem..

[99] Gao L., Tian Y., Gao W., Xu G. (2024). Recent Developments and Challenges in Solid-Contact Ion-Selective Electrodes. Sensors.

[100] Diamond D., Regan F. (1990). Resistance measurements as a simple diagnostic tool for ion-selective electrode performance. Electroanalysis.

[101] Kondratyeva Y.O., Solovyeva E.V., Khripoun G.A., Mikhelson K.N. (2019). Paradox of the Variation of the Bulk Resistance of Potassium Ion-Selective Electrode Membranes within Nernstian Potentiometric Response Range. Russ. J. Electrochem..

[102] Kondratyeva Y.O., Solovyeva E.V., Khripoun G.A., Mikhelson K.N. (2018). Non-constancy of the bulk resistance of ionophore-based ion-selective electrode: A result of electrolyte co-extraction or of something else?. Electrochim. Acta.

[103] Rafiee M., Abrams D.J., Cardinale L., Goss Z., Romero-Arenas A., Stahl S.S. (2024). Cyclic voltammetry and chronoamperometry: Mechanistic tools for organic electrosynthesis. Chem. Soc. Rev..

[104] Islam M.A., Mahbub P., Nesterenko P.N., Paull B., Macka M. (2019). Prospects of pulsed amperometric detection in flow-based analytical systems—A review. Anal. Chim. Acta.

[105] Fraher P., Clarke D.W. (1998). Fouling detection and compensation in Clark-type DOx sensors. IEEE Trans. Instrum. Meas..

[106] Kissinger P.T., Heineman W.R. (1983). Cyclic voltammetry. J. Chem. Educ..

[107] Peltola E., Sainio S., Holt K.B., Palomäki T., Koskinen J., Laurila T. (2018). Electrochemical Fouling of Dopamine and Recovery of Carbon Electrodes. Anal. Chem..

[108] Jang J., Cho H.-U., Hwang S., Kwak Y., Kwon H., Heien M.L., Bennet K.E., Oh Y., Shin H., Lee K.H. (2024). Understanding the different effects of fouling mechanisms on working and reference electrodes in fast-scan cyclic voltammetry for neurotransmitter detection. Analyst.

[109] Cen J., Vukas M., Barton G., Kavanagh J., Coster H. (2015). Real time fouling monitoring with Electrical Impedance Spectroscopy. J. Membr. Sci..

[110] Anseth R., Skeie N.-O., Waskaas M. (2018). Preliminary studies on monitoring fouling layers on a charged electrode using Electrical Impedance Spectroscopy. TM Tech. Mess..

[111] Fan Y., Huang Y., Linthicum W., Liu F., Beringhs A.O., Dang Y., Xu Z., Chang S.Y., Ling J., Huey B.D. (2020). Toward Long-Term Accurate and Continuous Monitoring of Nitrate in Wastewater Using Poly(tetrafluoroethylene) (PTFE)-Solid-State Ion-Selective Electrodes (S-ISEs). ACS Sens..

[112] De Marco R., Clarke G., Pejcic B. (2007). Ion-Selective Electrode Potentiometry in Environmental Analysis. Electroanalysis.

[113] Guo W., Ngo H.-H., Li J. (2012). A mini-review on membrane fouling. Bioresour. Technol..

[114] Müller-Steinhagen H., Malayeri M.R., Watkinson A.P. (2011). Heat Exchanger Fouling: Mitigation and Cleaning Strategies. Heat Transf. Eng..

[115] Chapman J., Regan F. (2012). Nanofunctionalized Superhydrophobic Antifouling Coatings for Environmental Sensor Applications—Advancing Deployment with Answers from Nature. Adv. Eng. Mater..

[116] Campuzano S., Pedrero M., Yáñez-Sedeño P., Pingarrón J.M. (2019). Antifouling (Bio)materials for Electrochemical (Bio)sensing. Int. J. Mol. Sci..

[117] Gu Y., Li Y., Wu Q., Wu Z., Sun L., Shang Y., Zhuang Y., Fan X., Yi L., Wang S. (2023). Chemical antifouling strategies in sensors for food analysis: A review. Compr. Rev. Food Sci. Food Saf..

[118] Liu B., Liu X., Shi S., Huang R., Su R., Qi W., He Z. (2016). Design and mechanisms of antifouling materials for surface plasmon resonance sensors. Acta Biomater..

[119] Jiang C., Wang G., Hein R., Liu N., Luo X., Davis J.J. (2020). Antifouling Strategies for Selective In Vitro and In Vivo Sensing. Chem. Rev..

[120] Lin P.-H., Li B.-R. (2020). Antifouling strategies in advanced electrochemical sensors and biosensors. Analyst.

[121] Turgut C., Newby B., Cutright T.J. (2004). Determination of optimal water solubility of capsaicin for its usage as a non-toxic antifoulant. Environ. Sci. Pollut. Res. Int..

[122] Villa F., Secundo F., Forlani F., Cattò C., Cappitelli F. (2021). Biochemical and molecular changes of the zosteric acid-treated Escherichia coli biofilm on a mineral surface. Ann. Microbiol..

[123] Donnelly B., Sammut K., Tang Y. (2022). Materials Selection for Antifouling Systems in Marine Structures. Molecules.

[124] Vonau W. (2010). Elektrochemische pH-Sensorik für SpezialanwendungenElectrochemical pH Sensors for Special Applications. tm–Tech. Mess..

[125] Trampert R. (2010). Email-Keramik-pH-Sensor im StahlverbundEnamel-Ceramic pH Sensors in a Steel-Compound. tm–Tech. Mess..

[126] Wencel D., Abel T., McDonagh C. (2014). Optical chemical pH sensors. Anal. Chem..

[127] Schulz V., Gerlach G., Günther M., Magda J.J., Solzbacher F. (2010). Piezoresistive pH Microsensors Based on Stimuli-Sensitive Polyelectrolyte HydrogelsPiezoresistive pH-Mikrosensoren auf der Basis stimuli-sensitiver polyelektrolytischer Hydrogele. tm–Tech. Mess..

[128] Gerlach G., Pechstein T. (2010). Der pH-Wert wird 100 Jahre alt. tm–Tech. Messen..

[129] Deng Z., Zhu R., Ma L., Zhou K., Yu Z., Wei Q. (2022). Diamond for antifouling applications: A review. Carbon.

[130] Li H., Cao J., Wei Q., Ma L., Zhou K., Yu Z., Zeng S., Zhu R., Yang W., Lin C.T. (2021). Antifouling nanoporous diamond membrane for enhanced detection of dopamine in human serum. J. Mater. Sci..

[131] Kumar R., Yang B., Barton J., Stejfova M., Schäfer A., Koenig M., Knittel P., Cigler P., Hirtz M. (2022). Diamond Surfaces with Clickable Antifouling Polymer Coating for Microarray-Based Biosensing. Adv. Mater. Interfaces.

[132] Long W., Li H., Yang B., Huang N., Liu L., Gai Z., Jiang X. (2020). Superhydrophobic diamond-coated Si nanowires for application of anti-biofouling’. J. Mater. Sci. Technol..

[133] Khorshidi B., Hosseini S.A., Ma G., McGregor M., Sadrzadeh M. (2019). Novel nanocomposite polyethersulfone-antimony tin oxide membrane with enhanced thermal, electrical and antifouling properties. Polymer.

[134] Zhang Z., Zheng J., Zhang Y., Zhang W., Li L., Cao Z., Wang H., Li C., Gao Y., Liu J. (2013). Anti-fouling in situ deposited antimony/nafion film electrode for electrochemical stripping analysis. Int. J. Electrochem. Sci..

[135] Yang W.J., Cai T., Neoh K.-G., Kang E.-T., Dickinson G.H., Teo S.L.-M., Rittschof D. (2011). Biomimetic anchors for antifouling and antibacterial polymer brushes on stainless steel. Langmuir ACS J. Surf. Colloids.

[136] Zouaghi S., Six T., Bellayer S., Moradi S., Hatzikiriakos S.G., Dargent T., Thomy V., Coffinier Y., Andre C., Delaplace G. (2017). Antifouling Biomimetic Liquid-Infused Stainless Steel: Application to Dairy Industrial Processing. ACS Appl. Mater. Interfaces.

[137] Yuan S., Wan D., Liang B., Pehkonen S.O., Ting Y.P., Neoh K.G., Kang E.T. (2011). Lysozyme-coupled poly(poly(ethylene glycol) methacrylate)-stainless steel hybrids and their antifouling and antibacterial surfaces. Langmuir ACS J. Surf. Colloids.

[138] Barish J.A., Goddard J.M. (2013). Anti-fouling surface modified stainless steel for food processing. Food Bioprod. Process..

[139] Magin C.M., Cooper S.P., Brennan A.B. (2010). Non-toxic antifouling strategies. Mater. Today.

[140] Halvey A.K., Macdonald B., Dhyani A., Tuteja A. (2019). Design of surfaces for controlling hard and soft fouling. Philosophical transactions. Ser. A Math. Phys. Eng. Sci..

[141] Shen G., Yang H., Hu Y., Zhang X., Zhou F., Li H., Hong K. (2022). Impact of Surface Roughness on Partition and Selectivity of Ionic Liquids Mixture in Porous Electrode. Nanomaterials.

[142] Sullivan T., O’Callaghan I. (2020). Recent Developments in Biomimetic Antifouling Materials: A Review. Biomimetics.

[143] Chambers L.D., Stokes K.R., Walsh F.C., Wood R. (2006). Modern approaches to marine antifouling coatings. Surf. Coat. Technol..

[144] Liu Z., Jiang T., Qin W. (2022). Polymeric Membrane Marine Sensors with a Regenerable Antibiofouling Coating Based on Surface Modification of a Dual-Functionalized Magnetic Composite. Anal. Chem..

[145] Jiang T., Qi L., Hou C., Fang S., Qin W. (2020). Self-Sterilizing Polymeric Membrane Sensors Based on 6-Chloroindole Release for Prevention of Marine Biofouling. Anal. Chem..

[146] Wang X., Liu T., Liang R., Qin W. (2024). Maintenance-free antifouling polymeric membrane potentiometric sensors based on self-polishing coatings. Analyst.

[147] Yebra D.M., Kiil S., Dam-Johansen K. (2004). Antifouling technology—Past, present and future steps towards efficient and environmentally friendly antifouling coatings. Prog. Org. Coat..

[148] Wardak C. (2013). 1-Hexyl-3-methylimidazolium hexafluorophosphate as new component of polymeric membrane of lead ion-selective electrode. Desalin. Water Treat.

